# Ten‐Eleven Translocation Family Proteins: Structure, Biological Functions, Diseases, and Targeted Therapy

**DOI:** 10.1002/mco2.70245

**Published:** 2025-07-01

**Authors:** Junzhi Liang, Xinni Na, Lingbo Meng, Lixia He, Ting Shu, Yuanyuan Fang, Bowen Zhang, Zhongyu Zhao, Cuishan Guo, Tingting Li, Zhijing Na, Da Li, Xue Xiao

**Affiliations:** ^1^ Center of Reproductive Medicine Department of Obstetrics and Gynecology Shengjing Hospital of China Medical University Shenyang China; ^2^ Department of Obstetrics and Gynecology Shengjing Hospital of China Medical University Shenyang China; ^3^ Department of Healthcare IT National Health Commission of the People's Republic of China National Institute of Hospital Administration Beijing China; ^4^ Department of General Internal Medicine VIP Ward Liaoning Cancer Hospital & Institute Shenyang China; ^5^ NHC Key Laboratory of Advanced Reproductive Medicine and Fertility (China Medical University) National Health Commission Shenyang China; ^6^ Key Laboratory of Reproductive Dysfunction Diseases and Fertility Remodeling of Liaoning Province Shenyang China; ^7^ Department of Gynecology and Obstetrics West China Second University Hospital Sichuan University Chengdu China; ^8^ Key Laboratory of Birth Defects and Related Diseases of Women and Children (Sichuan University) Ministry of Education, West China Second Hospital Sichuan University Chengdu China

**Keywords:** Biological functions, Demethylation, Diseases, Ten‐eleven translocation, Therapeutic strategies

## Abstract

Ten‐eleven translocation (TET) family proteins are Fe(II)‐ and α‐ketoglutarate‐dependent dioxygenases, comprising three family members: TET1, TET2, and TET3. These enzymes drive DNA demethylation by sequentially oxidizing 5‐methylcytosine to 5‐hydroxymethylcytosine, 5‐formylcytosine, and 5‐carboxylcytosine. Through these reactions, TET proteins remodel the epigenetic landscape and interact with transcription factors and RNA polymerase II to regulate gene expression, cell lineage specification, and embryonic development. Mutations and dysregulation of *TETs* have been associated with the pathogenesis of various diseases, including the nervous system, immune system, and metabolic diseases, as well as cancers. Therapeutic modulation of TETs may be an effective strategy for the treatment of these diseases. Here, we provide a comprehensive overview of the mechanisms by which TET proteins mediate DNA demethylation and detail their biological functions. Additionally, we highlight recent advances in understanding the molecular mechanisms linking *TET* dysregulation to disease pathogenesis and explore their potential as therapeutic targets. This review supplements the current understanding of the critical role of epigenetic regulation in disease pathogenesis and further facilitates the rational design of targeted therapeutic agents for diseases associated with mutations and dysregulation of *TETs*.

## Graphical abstract

TET family proteins play a critical role in DNA demethylation, and their mutations and dysregulation contribute to the onset of multiple diseases. With a deeper understanding of the molecular mechanisms of TET involvement in disease, drug therapy targeting TET proteins shows great potential, which provides insights into new therapeutic strategies.

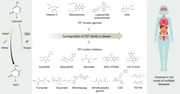

## 1 Introduction

Since 1942, when British developmental biologist Conrad Hal Waddington first introduced “epigenetics” to describe the interconnections between genotype and phenotype [1], researchers have never stopped exploring the impact of epigenetics on phenotype. DNA methylation, one of the most common epigenetic regulatory mechanisms, has been extensively studied [2, 3]. In addition, the opposite process of DNA methylation, known as DNA demethylation, has gained significance in recent years owing to its key role in various pathological processes.

DNA methylation begins with the addition of a methyl group to the fifth position of cytosine, catalyzed by DNA methyltransferase (DNMT), producing 5‐methylcytosine (5mC) [4]. These methylations interfere with the recognition of transcription factors and silence gene expression [5]. Although DNA demethylation has received particular attention as an important component of epigenetics, a clear understanding of this process remains lacking. Currently, DNA demethylation is believed to occur in two modes: passive and active demethylation. During active demethylation, ten‐eleven translocation family proteins (namely TET1, TET2, and TET3) play a crucial role. However, the protagonists of this story, the family of TET proteins, were not recognized to catalyze 5mC demethylation until 2009 [6]. This groundbreaking discovery was a key piece of the puzzle, revealing the mechanism of DNA methylation reversal and opening a new era in understanding mammalian epigenetics. Over the past decade or so, the studies on TET protein‐mediated DNA demethylation have developed rapidly, creating a dynamic methylome landscape. DNMTs catalyze the formation of 5mC, which is catalyzed by TET family proteins to sequentially generate 5‐hydroxymethylcytosine (5hmC), 5‐formylcytosine (5fC), and 5‐carboxycytosine (5caC) [3, 6, 7]. In mammals, DNA methylation and demethylation at the C5 position of cytosine are dynamic processes that are critical for controlling gene expression, cell fate reprogramming, and development [5].

TET plays an important role in the DNA methylation‐demethylation dynamic balance. Mutations or dysregulation of *TET* can lead to imbalances in the methylation landscape, resulting in the development of the nervous system, immune system, and metabolic diseases, as well as cancer [8, 9, 10, 11]. However, a comprehensive compendium of how TET mediates disease development and *TET*‐targeted therapeutic strategies is still lacking. In this review, we discuss the structure and functions of TET proteins, focus on available evidence on the involvement of TETs in disease, and summarize the cutting‐edge applied research on TET‐targeted therapy. Such comprehensive insights are pivotal for improving our understanding of disease pathogenesis and developing new drugs for disease treatment.

## 2 Structural Features of TET Proteins

TET proteins were named “ten‐eleven translocations” after the discovery of *TET1* in a chromosomal translocation process that fused the mixed lineage leukemia gene on chromosome 11 with the *TET1* located on chromosome 10 [12, 13]. Later, *TET1*, *TET2*, and *TET3* were successively demonstrated to be located on chromosomes 10q21.3, 4q24, and 2p13.1, respectively [12, 14, 15].

TET proteins are Fe(II) and α‐ketoglutarate (α‐KG)‐dependent dioxygenases with multiple structural domains at a size of approximately 180–230 kDa [16, 17]. In mammals, all three TET proteins are characterized by the presence of a conserved double‐stranded β‐helix (DSBH) structural domain and cysteine‐rich structural domain, which together form the core catalytic region of the C‐terminal [18, 19]. Structural studies show that the DSBH structural domain brings together Fe(II), α‐KG, and 5mC for oxidation, while the cysteine‐rich structural domain surrounds DSBH to stabilize the overall structure and sustain the reaction [17, 20]. Strikingly, a low‐complexity domain in the DSBH domain was recently found to regulate TET activity and inhibit aberrant 5mC oxidation [21, 22]. In addition to their catalytic domains, TET1 and TET3 have an N‐terminal Cys‐X‐X‐Cys (CXXC) zinc finger domain that mediates interactions with various nuclear components, thus influencing developmental processes [16, 23]. Whereas TET2 underwent a chromosomal inversion event during evolution, the exon containing the CXXC structural domain was segregated as a separate gene encoding IDAX (also known as CXXC4) [18, 19]. These CXXC domains can bind unmethylated cytosine–guanine (CpG) and play an important role in the accurate targeting of TET to the genome [24]. Notably, CXXC also plays an important role in the noncatalytic function of TET [25]. Taken together, these unique structural domains endow TET proteins with the ability to precisely engage in methylation, thereby playing a unique role throughout life.

Although the TET family proteins exhibit structural and functional similarities and share catalytic activities, genetic studies have strongly suggested that these TET proteins are not simple functional duplicates. Additional mechanistic studies will help us better understand this mystery.

## 3 Mechanisms of DNA Methylation and TET‐Dependent Demethylation

**FIGURE 1:**
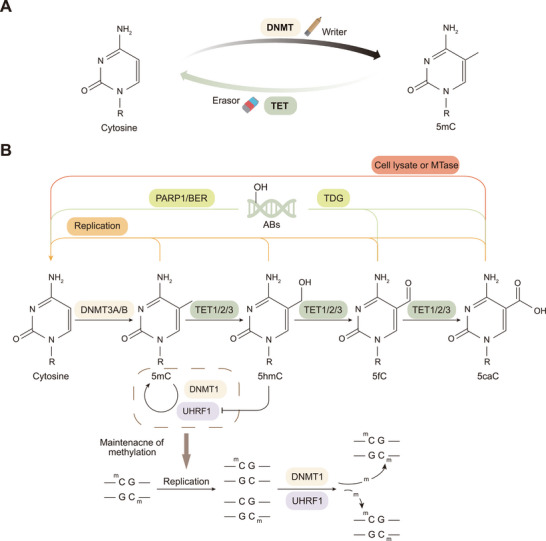
Mechanisms of DNA methylation and TET‐dependent demethylation. (A) DNMT acts as a DNA methylation writer to add methyl groups to cytosine, and TET proteins act as methylation erasers involved in the precise regulation of the DNA methylation landscape. (B) Cytosine on DNA is converted to 5mC under the catalytic action of DNMT3A/B/C (DNMT3L is involved in regulation). DNMT1 and its accessory factor UHRF1 maintain the methylation of double strands in DNA replication. The demethylation process consists mainly of passive and active forms. Passive demethylation refers to the failure of maintenance of methylation by DNA replication, which eventually manifests itself as 5mC dilution, whereas active demethylation is an ongoing process of research. We summarize the current understanding of the mechanism of three DNA demethylation pathways mediated by TET proteins, including: (1) TDG converts these 5fC and 5caC bases to transient abasic sites and DNA single strand breaks, followed by DNA damage repair by PARP1 in concert with BER proteins; (2) TET‐catalyzed generation of 5hmC inhibits UHRF1 and DNMT1, leading to a failure in maintenance of methylation. This results in DNA breaks, followed by repair of DNA damage by PARP1 in concert with BER proteins; (3) cell lysate and MTases are involved in the direct decarboxylation of 5caC. Abbreviations: DNMT, DNA methyltransferase; TET, ten‐eleven translocation; 5mC, 5‐methylcytosine; MTases, S‐adenosylmethionine‐dependent DNA methyltransferases; PARP1, poly(ADP‐ribose) polymerase 1; BER, base excision repair; ABs, abasic sites; TDG, thymine DNA glycosylase; DNMT3A/B/C, DNA methyltransferase 3A/B/C; DNMT3L, DNA methyltransferase 3‐like protein; 5hmC, 5‐hydroxymethylcytosine; 5fC, 5‐formylcytosine; 5caC, 5‐carboxylcytosine; UHRF1, ubiquitin‐like containing PHD and RING finger domains 1.

DNA methylation is dynamically regulated through two opposing processes: establishment/maintenance and demethylation. Establishment and maintenance are mediated by DNMTs, which act as methylation writers, whereas demethylation is driven by TET proteins, which function as erasers to precisely regulate the methylation landscape (Figure 1A) [21]. In mammals, the methylation product 5mC has far‐reaching effects on genome stability, gene expression, genomic imprinting, and X chromosome inactivation [26, 27]. Three main DNMTs, namely, DNMT3A, DNMT3B, and DNMT1, are responsible for DNA methylation and play different roles in the establishment and maintenance of DNA methylation patterns [28]. DNMT3A and DNMT3B are mainly responsible for adding methyl groups to unmethylated CpG sites, thereby establishing DNA methylation patterns during early development [29]. DNMT1 and its accessory protein, ubiquitin‐like containing PHD and RING finger domains 1 (UHRF1), maintain the methylation pattern after DNA replication by recognizing the hemimethylated CpG site and catalyzing the methylation of the daughter strand [4, 17, 27]. Additionally, two DNMT homologs, DNMT2 and DNMT3L, are considered noncanonical members of the DNMT family, as they lack the catalytic DNMT activity [30].

The demethylation process can be divided into passive demethylation and active (TET‐dependent) demethylation. Among them, passive DNA demethylation refers to the process of cell division in which DNA replication results in the failure to maintain the DNA methylation pattern, which ultimately manifests as a dilution of 5mC [16]. Active demethylation mainly occurs through the oxidation of TET family proteins, which convert 5mC to 5hmC, 5fC, and 5caC to form demethylation intermediates, which are subsequently recognized by thymine DNA glycosylase (TDG) and repaired through the dependent base excision repair (BER) pathway, ultimately reverting to unmodified cytosine (Figure 1B) [31, 32, 33, 34]. Both the N‐terminal and C‐terminal catalytic domains of TET contain specific TDG‐interacting domains, which are essential for the DNA demethylation process jointly mediated by TET and TDG [35]. The oxidation product of the TET protein, 5hmC, can actively promote DNA demethylation by inhibiting the binding of the UHRF1/DNMT1 complex to DNA, thereby leading to methylation maintenance failure [36]. In addition, recent studies have shown that the TET‐catalyzed generation of 5caC can be converted to an unmodified cytosine by a direct decarboxylation process catalyzed by specific conditions or enzymes, further revealing the complexity of DNA demethylation [37, 38].

Although TET proteins were initially thought to play the only dominant role in DNA demethylation, as research progressed, it was found that demethylation may involve the synergistic action of multiple pathways, of which TET proteins constitute only one link. In addition to TET proteins, other repair mechanisms and metabolic pathways may also play important roles in converting 5mC to cytosine. Therefore, further study of these mechanisms could help reveal the important role of DNA demethylation in cell reprogramming, development, and evolution.

## 4 Biological Functions of TET

Active DNA demethylation plays a role in various biological processes, including DNA methylation reprogramming during preimplantation and primordial germ cell (PGC) development, and maintenance and differentiation of embryonic stem cells (ESCs). DNA methylation reprogramming refers to the genome‐wide removal of epigenetic marks from the parental genome through extensive DNA demethylation, followed by remethylation to establish the epigenetic state of the early embryo [39]. Although DNA methylation reprogramming has been found to occur in mammalian development for more than three decades, it was not until the advent of whole‐genome bisulfite sequencing that DNA methylation kinetics could be assessed with single‐base resolution [40, 41]. With the advancement of precise assays to assess DNA methylation levels at genetic loci, tremendous progress has been made in understanding these epigenomic reprogramming processes. Epigenetic reprogramming leads to global genome hypomethylation and severe loss of genetic memory, which underlies the acquisition of pluripotency, where cell fate is redetermined [42]. Thereafter, methylation proceeds progressively, and cells gradually differentiate to promote the development of various systems. The dynamic regulation of DNA methylation patterns, including reprogramming, is critical for mammalian development and differentiation.

### 4.1 DNA Methylation Reprogramming: The Core Role of TET Proteins

**FIGURE 2:**
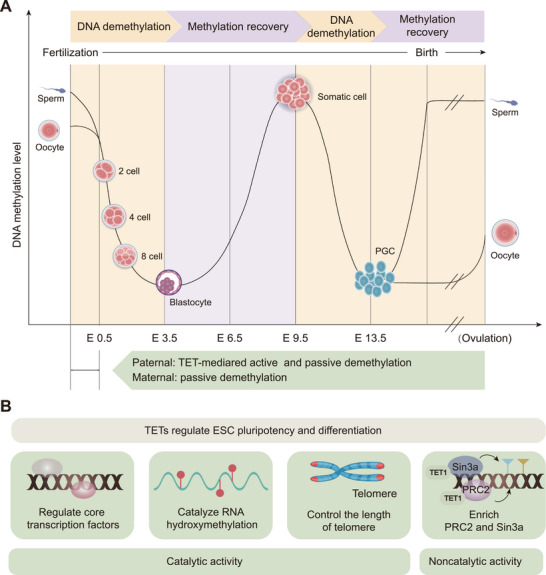
Biological functions of TET. (A) TET proteins are involved in two DNA methylation reprogramming processes. In the first reprogramming, which occurs during the early stages of fertilization, the paternal and maternal genomes undergo independent demethylation processes. The paternal genome undergoes active and then passive demethylation, whereas the maternal genome is passively demethylated in a DNA replication‐dependent manner until the level of DNA methylation is minimized at the blastocyst stage. The second reprogramming occurs during the establishment of PGCs. Methylation gradually recovers after the blastocyst stage. The restored somatic cells are demethylated at the E9.5–E13.5 stage, mediated by TET1 and TET2, and reach the lowest level of DNA methylation around day E13.5. PGCs differentiate to form sperm or eggs in different sexes and undergo different methylation regulation during differentiation. (B) TET proteins are able to regulate ESC pluripotency and differentiation through both catalytic and noncatalytic functions. TET proteins rely on their catalytic activity to regulate ESC pluripotency and differentiation by regulating core transcription factors, catalyzing hydroxylation of RNA, and controlling chromosome length. Meanwhile, TET also enriches PRC2 and Sin3a to participate in the regulation of ESCs through noncatalytic functions. Abbreviations: E, embryonic day; ESC, embryonic stem cell; PGC, primordial germ cell; PRC2, polycomb repressive complex; TET, ten‐eleven translocation.

In the mammalian genome, 5mC is located predominantly in CpG dinucleotides, with most CpGs in the genome being methylated [43]. These methylations provide a molecular memory that ensures faithful adherence to transcriptional programs during mammalian development [42]. However, during the mammalian life cycle, there are two incidences of methylation reprogramming: the first occurs during the early embryo stage after fertilization, and the second occurs during the establishment of PGCs (Figure 2A) [25, 44].

#### 4.1.1 First Reprogramming—Demethylation of Paternal and Maternal Genomes in Preimplantation Fertilized Zygotes

The first reprogramming typically occurs approximately 3–4 days after fertilization, when the genomes of paternal and maternal origins within the fertilized zygotes undergo erasure of methylation imprints and the subsequent establishment of embryonic methylation patterns by different mechanisms [45].

In the first reprogramming, the paternal genome undergoes a complex process of epigenetic remodeling. Before fertilization, the mature sperm genome presents the highest level of DNA methylation among all mammalian cell types, with approximately 90% of the CpG being methylated [45]. After fertilization, the paternal genome is first actively demethylated, followed by passive loss, and reaches a minimum at the blastocyst stage [46]. Beginning 6 h after fertilization when the paternal genome undergoes active DNA demethylation, this process results in active methylation deprivation of most of the bulk of the methylation except imprinting control regions and some retrotransposons [44, 47, 48]. This demethylation mechanism is catalyzed by TET3, and the intermediates generated such as 5hmC, 5fC, and 5caC, are diluted by DNA replication [49, 50, 51]. This conclusion has been supported by several studies. Immunostaining analyses revealed that TET3 and 5hmC are predominantly located in the paternal pronucleus of fertilized oocytes [47, 50, 52]. However, in *TET3*‐deficient fertilized zygotes from conditional knockout mice, 5mC to 5hmC conversion of the paternal genome failed and the level of 5mC remained unchanged, demonstrating the dominant role of TET3 in this active demethylation process [50]. Following this dramatic transition, DNA methylation levels in the paternal genome of zygotes appear to be passively lost until the blastocyst stage, when methylation is at its lowest point [53]. Recent evidence suggests that passive demethylation may also partially be facilitated by TET‐mediated hydroxylation [53]. Interestingly, DNA demethylation has been reported to precede DNA replication and TET3‐mediated oxidation, suggesting that there may be a TET3‐independent demethylation mechanism [54]. This finding implies that epigenetic reprogramming is a complex process, and further research is needed to identify the key factors involved in this fascinating process. In conclusion, the prevailing view is that DNA replication and TET3‐mediated DNA demethylation are mainly responsible for paternal DNA demethylation [55].

Global DNA methylation levels in the maternal genome are lower than those in the paternal genome of early fertilized zygotes [45]. Despite being in the same environment, the maternal genome is not affected by TET3‐mediated active demethylation but is passively demethylated in a DNA replication‐dependent manner [56]. In contrast to the rapid demethylation of the paternal genome postfertilization, the maternal genome undergoes progressive demethylation in the subsequent cell cycle, up to the morula stage [57]. This process raises the question of how the maternal genome protects 5mC from TET3‐mediated oxidation and undergoes passive demethylation in a DNA replication‐dependent manner, a phenomenon that has attracted considerable attention in recent years. The maternally inherited Stella (also known as PGC7 or DPPA3) protein plays an indispensable role in this process [58, 59], as demonstrated by the fact that Stella knockdown facilitates oxidation of 5mC to 5hmC [49, 59]. Mechanistically, Stella inhibits TET3 activity by binding to chromatin containing dimethylated histone H3 lysine 9, thereby protecting 5mC from TET3‐mediated demethylation [60]. Moreover, Stella protects the unique oocyte epigenome by preventing DNMT1‐ and UHRF1‐mediated aberrant DNA methylation, promoting passive demethylation in a DNA replication‐dependent manner [59]. Notably, TET3 also contributes to the demethylation of the maternal genome, although to a lesser extent than in the paternal genome [55]. In conclusion, by inhibiting TET and disrupting methylation maintenance, the maternal genome undergoes passive demethylation in a DNA replication‐dependent manner.

The methylation landscapes of the paternal and maternal genomes in early zygotes have long been explored to elucidate the complex and sophisticated regulatory codes of embryonic development. The ongoing advancement of methylation detection tools has enabled us to peek into the mysteries and marvel at them. Passive demethylation in a DNA replication‐dependent manner is the primary mode of paternal and maternal genome demethylation in early embryos [55]. However, DNA methylation is highly dynamic and complex during mammalian embryonic development; a clear overview of this process is lacking, and further research is needed for a more precise understanding.

#### 4.1.2 Second Reprogramming—DNA Demethylation in PGC

PGCs in mice are precursors of sperm and oocytes [61], and various attempts have been made to explore their genome‐wide epigenetic reprogramming processes. The current understanding of methylation reprogramming in PGCs is based on studies of early mouse embryos. In mice, the global erasure of DNA methylation in PGC consists of two consecutive steps. The first stage is at embryonic day 6.5–9.5 (E6.5–E9.5) when both the TET protein and 5hmC are present at very low levels and when DNA demethylation relies primarily on the passive dilution of 5mC during replication [62]. Over 70% of the CpGs are demethylated during this phase; however, certain regions, including imprinted sites and germline‐specific genes, maintain DNA methylation [56, 63, 64]. Understanding how these regions are protected from the first wave of DNA demethylation remains essential for further investigation [63]. These remaining unmethylated portions are removed by TET1 and TET2 during the second stage, embryonic days 9.5–13.5 (E9.5–E13.5) [40, 63]. Unexpectedly, after demethylation, PGCs exhibit hypomethylation, but with a small degree of methylation retained, including at intracisternal A particles and some regions of long interspersed nuclear elements and short interspersed elements [64]. This two‐stage DNA demethylation is thought to prevent hypogonadism and sterility caused by the premature differentiation of PGC into the germ line [65].

The DNA methylation landscape is completely reshaped following two global demethylation cycles. Both reprogramming processes are global; however, DNA methylation persists at the end of both processes, and how demethylation escape is achieved has long been elusive. Nevertheless, this finding suggests the intriguing possibility of intergenerational or even transgenerational epigenetic inheritance [40].

### 4.2 TET and ESC Pluripotency and Differentiation

*TET* expression is a dynamic process that occurs throughout the life cycle. At the mouse ESC (mESC) stage, *TET1* and *TET2* are highly expressed, whereas *TET3* is almost undetectable [66, 67]. With the development and differentiation of cells, *TET1* levels begin to decrease, whereas *TET2* and *TET3* are widely expressed in various tissues, including hematopoietic and neuronal tissues [16, 68]. Despite low levels, TET3 still functions as a catalyst for 5hmC in mESCs [69, 70]. The three TET proteins functionally overlap, but each protein plays a unique role [71]. *TET* knockout studies in ESCs and mice have demonstrated that all three TET proteins are involved in regulating embryonic development and fetal organogenesis (Table 1) [72]. ESCs lacking a single TET enzyme do not affect pluripotency but exhibit differential differentiation defects, whereas deleting all three *TET* genes severely impairs normal ESC lineage commitment [73]. Mice lacking TET1 are viable but smaller and have significantly reduced female germ cell numbers and fertility [74, 75]. Like TET1, TET2 is dispensable for embryonic development and adult mice are viable and fertile. However, TET2 deficiency promotes the formation of myeloid malignancies [76, 77]. In contrast, combined deficiency of TET1 and TET2 results in developmental defects and increased perinatal mortality [66]. The deficiency of all three TET enzymes fails in progenitor embryonic development, leading to early embryonic lethality, highlighting the importance of TET enzymes in embryogenesis [78, 79].

**TABLE 1:** Studies exploring the role of TET in early embryonic development and differentiation by deleting *TET*.

Model	Depletion strategy	Phenotypic changes	References
mESC	*TET1*, *TET*2, *TET3* shRNA KD	*TET1* KD reduced Nanog, Oct4, and Sox2 expression, impaired mESC self‐renewal and maintenance	[3]
mESC	*TET1*, *TET*2, *TET*3 siRNA KD; *TET1*, *TET2* shRNA KD	Skewed differentiation into the endoderm–mesoderm lineage in embryoid bodies; skewed to trophectoderm	[67]
mESC	*TET1* KO	Displayed skewed differentiation toward trophectoderm in vitro; exhibited a slightly smaller body size at birth	[75]
mESC	*TET1* KO	Reduced female germ‐cell numbers and fertility; univalent chromosomes and unresolved DNA double‐strand breaks in oocytes	[74]
mESC	*TET3* KO	Impaired neural conversion, with skewing toward cardiac mesoderm	[80]
mESC	*TET1*/2 DKO	Reduced 5hmC levels and promoted global hypermethylation	[66]
mESC	*TET1*, *TET2* KO	Deletion of *TET2* resulted in reduced genome‐wide DNA hydroxymethylation and increased enhancer DNA methylation levels	[81]
mESC	*TET1/2/3* TKO	Poorly differentiated TKO embryoid bodies and teratomas	[70]
mESC	*TET1/2/3* TKO; *TET1/2* DKO; *TET3* KO	TET‐mediated RNA hydroxymethylation was found to reduce the stability of crucial pluripotency‐promoting transcripts	[82]
mESC	*TET1*, *TET2* KD; *TET1*, *TET2*, *TET3* KO	Exhibited short telomeres and chromosomal instability, concomitant with reduced telomere recombination	[83]
mESC	*TET1/2/3* TKD	Cells with disrupted signaling, leading to skewed whole‐embryo mutant gastrulation	[79]
mESC	*TET1*, *TET2*, *TET3* KD	5hmC levels were unaffected by *TET1* or *TET2* depletion but significantly reduced upon *TET3* depletion, directly promoting R‐loop formation	[69]
mESC	*TET1^m/m^* and *TET1* KD	Noncatalytic functions of TET1 suppress aberrant differentiation of ESCs toward the trophectoderm lineage	[73]
hESC	*TET1/2/3* TKD	Complete inability to form teratomas and impaired induction of key early differentiation genes upon spontaneous embryoid body differentiation	[84]
hESC	*TET2* KD	No effect on the pluripotent markers of hESCs; skews spontaneous differentiation of hESCs toward neuroectoderm	[85]
Mice	*TET1^m/m^* and *TET1* KD	*TET1^−/−^*, but not *TET1^m/m^*, embryos exhibit markedly fewer somites, weigh less, and are smaller in size	[73]
Mice	*TET2* KO	Caused myeloid malignancies	[76]
Mice	*TET2* KO	Expansion of HSPCs and the development of myeloid and lymphoid disorders	[77]
Mice	*TET1/2* DKO	Most double mutant mice died perinatally; surviving *TET1/TET2* deficient mice were fertile with females having smaller ovaries and reduced fertility	[66]
Mice	*TET1/2/3* TKO	Gastrulation defects	[78]

Abbreviations: 5hmC, 5‐hydroxymethylcytosine; DKO, double knockout; ESC, embryonic stem cell; hESC, human embryonic stem cell; HSPC, hematopoietic stem and progenitor cell; KD, knockdown; KO, knock out; mESC, mouse embryonic stem cell; OCT4, octamer‐binding transcription factor 4; shRNA, short hairpin RNA; siRNA, small interfering RNA; Sox2, SRY‐box transcription factor 2; TET, ten‐eleven translocation; TET1^m/m^, TET1 catalytic mutant; TKO, triple knockout.

ESCs are derived from the ectoderm of mammalian blastocysts and are highly specialized pluripotent cells capable of self‐renewal or differentiation into virtually any cell type [86]. ESC fate decisions (determining whether to remain pluripotent or undergo differentiation) must be tightly controlled for normal development. TETs play a unique role in the pluripotency and differentiation of ESCs through their catalytic and noncatalytic functions through multiple pathways (Figure 2B).

ESCs maintain their pluripotent state mainly by regulating core transcription factors, such as octamer‐binding transcription factor 4 (OCT4), SRY‐box transcription factor 2 (SOX2), and nanog homeobox (NANOG) [87, 88]. TET proteins interact with these core transcription factors and regulate ESC pluripotency and differentiation. TET1 has been reported to play an important role in the self‐renewal and maintenance of ESCs by binding to the NANOG promoter and maintaining its hypomethylation status [3]. However, these transcription factors can inversely affect *TET* expression. In ESCs, the promoters of the *TET1* and *TET2* genes can be bound and activated by pluripotent transcription factors, and depletion of OCT4 and SOX2 leads to the downregulation of mRNAs for *TET1* and *TET2*, and rapid differentiation of ESCs [67, 89, 90]. Additionally, recent findings have revealed that TET proteins catalyze RNA hydroxymethylation in ESCs, which reduces the stability of important pluripotency‐promoting transcripts, thereby ensuring orderly ESC differentiation [82]. Furthermore, TET proteins regulate pluripotency by controlling telomere length [83]. Telomeres maintain genomic stability, which is essential for self‐renewal and pluripotency of ESCs [91, 92]. *TET1* and *TET2* depletion leads to telomere shortening and chromosomal instability in ESCs [83]. The specific mechanism of this process is that *TET* depletion or deficiency increases DNMT3B and decreases 5hmC levels, ultimately leading to elevated levels of sub‐telomeric methylation, resulting in telomere shortening and chromosomal instability [83]. These findings highlight that TET enzymes play an important role in telomere maintenance and chromosomal stability in ESCs.

Recently, the catalytic activity of TET1 has been proposed to be more important during late gestation and postnatal development, whereas its noncatalytic function is more critical during ESC differentiation and early embryonic development [73]. By constructing TET1 catalytically inactive (*TET1^m/m^*) and knockout (*TET1^−/−^*) ESCs and mice, Chrysanthou et al. [73] reported that TET1, through its noncatalytic role, can promote polycomb repressive complex (PRC2) (for lysine 27 on histone H3 [H3K27] trimethylation) and Sin3a complex (for H3K27 deacetylation) enrichment on bivalent gene promoters, thereby increasing H3K27 trimethylation and deacetylation and repressing developmental gene expression. These findings suggest that the noncatalytic function of TET1 is essential for the proper regulation of the ESC gene expression program as well as stem cell identity and plasticity. These effects were not related to the DNA demethylation activity of TET1, but dependent on its protein structure and interactions.

TET proteins play important roles in ESC differentiation. The knockdown of *TET1* leads to alterations in the differentiation tendency of ESCs, which is manifested mainly as skewed differentiation toward the trophectoderm and mesendoderm [67]. The deletion of *TET1* decreases the expression of the Nodal antagonist Lefty, which enhances Nodal signaling and promotes skewed differentiation [67]. The deletion of *TET2* in mESCs results in increased hypermethylation and delayed gene induction during the early stages of differentiation [81]. Specifically, *TET2* deficiency induces biased differentiation of mESCs toward the neural ectoderm [67]. This finding is consistent with the results in hESCs, in where the knockdown of *TET2* alters promoter methylation of key regulatory genes, thereby favoring neural ectodermal differentiation [85]. Lack of *TET3* showed impaired neural conversion and was concomitantly skewed toward a cardiac mesodermal fate [80]. *TET1/2/3* triple‐knockout (TKO) mESCs presented an impaired overall differentiation capacity, which manifested as poorly differentiated embryoid bodies and teratomas [70], consistent with the impaired differentiation in TKO hESCs [84]. These findings suggest that defects in *TETs* can bias differentiation toward certain lineage‐specific cells, leading to the development of aberrant differentiation.

In summary, TET proteins influence mammalian developmental processes by regulating core transcription factors, RNA hydroxymethylation, telomere length, and the PRC2 and Sin3a complexes. These findings establish that TET precisely mediates ESC development and differentiation via its catalytic and noncatalytic functions and demonstrate the critical role of these enzymes in embryonic development and differentiation.

## 5 TET Proteins and Diseases

**FIGURE 3:**
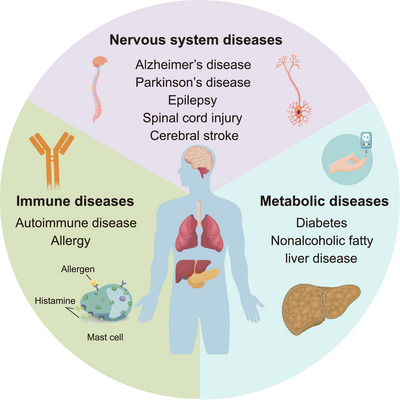
Dysregulation of TET proteins are widely involved in the development of noncancer diseases, including nervous system, immune, and metabolic diseases. Although TET predominantly acts as a cancer suppressor, it can also act as an oncogenic factor. *Abbreviation*: TET, ten‐eleven translocation.

The DNA methylation landscape is dynamically regulated by DNMT and TET [72]. This dynamically changing DNA methylation landscape subtly regulates gene expression through stepwise remodeling of DNA methylation, which mediates a variety of physiological and pathological processes [93]. Over the past two decades, mutations and dysregulation of *TET* are associated with the pathogenesis of a wide range of human diseases, including noncancer (nervous system, immune system, and metabolic diseases) (Figure 3) as well as cancer diseases.

### 5.1 TET and Nervous System Diseases

TET family proteins are involved in neurodevelopment and homeostasis maintenance through dynamic regulation of 5hmC. The abnormal function of TET proteins disrupts 5hmC regulation, contributing to neurological disorders such as Alzheimer's disease (AD), Parkinson's disease (PD), epilepsy, spinal cord injury (SCI), and cerebral stroke.

#### 5.1.1 TET and AD

AD is a neurodegenerative disorder affected by multiple regulators, including epigenetic and genetic factors [94, 95]. Recent studies have suggested that a dynamic imbalance of 5hmC plays a key role in the pathological process of AD [96]. It has been shown that overall levels of 5hmC are decreased in AD brain tissue [97, 98].

Defective TET enzymatic activities and content in the brain tissues of patients with AD are considered the main reasons for the inadequate frequency of 5hmC, whereas the activation of functional domains of TET proteins alleviates the progression of neurodegenerative processes [97]. The additional administration of methyl donors is also beneficial for the reduction of Tau phosphorylation, as well as the improvement of learning and motor phenotypes [99]. Recently, Armstrong et al. [95] conducted a cohort study of patients with early‐onset AD. Through gene‐wise burden analysis and a portrayal of 5hmC patterns in human postmortem brain tissues, they reported that TET1 loss‐of‐function variants contributed to AD‐associated pathology, accompanied by altered contents of 5hmC among regulatory genes in AD. Based on data from AD model mice, a decrease in TET1 and dysregulation of methylation led to remarkable impairment in the expression of 40 genes related to glial cell activation and neurosynaptic formation [95]. Similarly, Cochran et al. [100] showed that *TET2* noncoding and loss‐of‐function variants predominantly accumulated within regulatory DNA fragments related to AD. Overexpression of *TET2* in the hippocampal neurogenic tissues of adult mice increased 5hmC and rescued the age‐related decline in neurogenesis, especially learning and memory capabilities [101]. Additionally, emerging studies have proposed that neuroinflammation and the proinflammatory cytokine interleukin 6 (IL‐6), which are crucial pathological hallmarks of AD, could potentially inhibit *TET3* expression in a Janus kinase 2 (JAK2)/signal transducer and activator of transcription 3 (STAT3)‐dependent manner, leading to the downregulation of Neurogenic differentiation 1 in neural stem cells [102]. Blocking JAK2/STAT3 or increasing *TET3* expression exerts a positive role in hippocampal neurogenesis and cognitive function reservation in AD model mice [102].

Taken together, these results indicate that TET protein deficiency is widely associated with AD pathology and severity. Existing research is mostly limited by the sample size of human brain tissue where efforts should be focused, and future attempts are needed to extend molecular‐level mechanical discoveries to a wider population to assess potential approaches for AD diagnosis or treatment.

#### 5.1.2 TET and PD

The incidence rate of PD ranks second on the list of common neurodegenerative disorders worldwide, following AD [103]. Recent perspectives suggest that the pathological hallmarks of PD, including the accumulation of Lewy bodies and the deposition of α‐synuclein proteins, may be potentially related to gene mutations and epigenetic alterations [104, 105].

Studies have shown that epigenetic and transcriptional levels of *TET2* are significantly upregulated in neurons of PD patients, leading to an abnormal increase in hydroxymethylation levels in the enhancer region, which may exacerbate disease progression by triggering aberrant neuronal function and immune‐inflammatory responses [106]. In contrast, microglia‐mediated neuroinflammation could be reduced by inhibiting TET2 activity, and nigrostriatal dopaminergic neuron loss was prevented [106, 107]. These findings suggest that TET2 is both a key regulator and a potential therapeutic target in the pathomechanism of PD and that targeted inhibition of TET2 may provide a new strategy for delaying or treating PD.

#### 5.1.3 TET and Epilepsy

Epilepsy is a series of disorders featuring disturbed synaptic transmission and a balanced state between excitatory and inhibitory signals [108, 109]. Maintenance of DNA methylation is influenced by DNMT and TET, which are responsible for the function and activity of inhibitory cortical interneurons via the endocytosis of neurotransmitters [110]. According to a descriptive cross‐sectional pilot study, global DNA methylation markers are decreased in the hippocampus, whereas there is an increase in the neocortex in adults with temporal lobe epilepsy and children with febrile seizures [111]. Based on current research progress, overactivation of TET‐dependent DNA demethylation has been associated with cell proliferation and disturbance of excitation and inhibition in epilepsy. In patients with drug‐resistant epilepsy, *TET2* expression in the temporal lobe cortex is considerably elevated, especially within vascular regions [112]. *TET2* depletion results in the inhibition of ABCB1 in the blood–brain barrier, a dominant pathological gene in epilepsy [112]. In addition, there is a transient induction of TET‐dependent demethylation in status epilepticus accompanied by a sharp upregulation of Gadd45b in newly generated neurons in the subgranular zone, leading to an increased rate of cell proliferation [113]. Zybura‐Broda et al. [114] further explained that increased Gadd45b accumulated within the region of gene promoters in matrix metalloproteinase‐9 (MMP‐9) leads to excess expression, contributing to the progression of epilepsy. Epilepsy‐related aberrant neuronal activity may be attributed to a combination of factors, suggesting a need for a deeper understanding of the complex DNA methylation patterns rather than solely focusing on TET in the current model [115, 116]. Nevertheless, interventions targeting CpG demethylation associated with TET proteins may improve the related symptoms and pathologies.

#### 5.1.4 TET and SCI

DNA methylation and demethylation are of great importance in the regulation and induction of axonal regeneration following SCI [117]. In the acute and subacute stages of SCI, global levels of 5hmC are markedly increased, which is influenced by the overexpression of *TETs* [118, 119]. Davaa et al. [120] proposed that the maintenance of enhanced levels of TET proteins is a positive element in the recovery of locomotor, memory, and cognitive functions, which partly explains the therapeutic efficacy of exercise training in SCI. Petrie et al. [121, 122] further investigated the impacts of electrical‐induced skeletal muscle exercise on the activation of 38 gene sets related to systemic metabolism and muscle strength, notably, the demethylation of the main transcription factor PGC‐1α. Similarly, Hong et al. [119] induced long‐term upregulation of *TET* expression in SCI rats through the administration of ascorbic acid, suggesting a potential association between TET‐dependent DNA demethylation and axonal sprouting within the lesion. After peripheral axotomy of the dorsal root ganglia, an increase in *TET3* was observed along with the upregulation of 5hmC within a large set of regeneration‐associated genes [123]. However, it remains controversial whether high 5hmC levels and overexpression of *TET* are compensatory repair responses to SCI or subsequent injury factors. Demethylation of the promoter region of G protein‐coupled receptors (GPRs) such as GPR151 and CXC chemokine receptor 3, after SCI enhances neuropathic pain via the map kinase pathway [124, 125]. Additionally, Sun et al. [126] reported that inhibiting *TET2* overexpression after SCI resulted in the downregulation of genes involved in cell death‐related genes, thereby reducing the area of necrosis. Given the alterations in TET proteins and 5hmC global levels within the lesion cavity after SCI, proper DNA methylation states do play a crucial role in the maintenance of regenerative capacity in SCI. Although potent supplements or interventions that achieve subsequent and constant DNA demethylation remain a novel perspective for triggering axonal regeneration, whether the current conclusion can be directly and widely applied in clinical practice requires further discussion and detailed verification.

#### 5.1.5 TET and Cerebral Stroke

Alterations in DNA methylation greatly influence the pathogenesis, development, and post‐stroke recovery of ischemic or hemorrhagic brain injury [127, 128]. The neuroprotective roles of TET proteins and 5hmC have been previously highlighted [129]. Genome‐wide profiling of 5hmC in brain tissues with ischemic injury revealed an altered pattern of demethylation in regions associated with neuronal morphogenesis and synaptogenesis [130]. A marked decrease in the global levels of 5hmC and TET proteins was observed within 24 h after intracerebral hemorrhage, which lasted for approximately 72 h. Hypomethylation hallmarks are mainly located in genes that contribute to cell death after brain injury [131]. TET2‐mediated 5hmC modifications play a crucial role in hydroxymethylation of the brain‐derived neurotrophic factor promoter, improving the pathology of infarction after ischemic injury [130]. Focal ischemic lesions also trigger the overactivation of TET3, catalyzing the enriched formation of 5hmC. Elevated demethylation considerably promotes the expression of diverse genes associated with DNA repair and antioxidant responses, thus providing potent protection against ischemic injury [132]. In contrast, neonatal hypoxic‐ischemic brain injury markedly decreases *TET2* expression, which is regulated by enhanced levels of miR‐210. A defect in *TET2* led to the secretion of proinflammatory cytokines in a nuclear factor kappa B (NF‐κB)/histone deacetylase 2/3/IL‐1β manner, explaining neuroinflammation caused by microglia from ischemic lesions [133]. Additionally, mitochondrial *TET2* expression is altered in response to cerebral ischemia, increasing the abundance of 5hmC and the regulation of mitochondrial genes [127]. Although mitochondrial dysfunction plays an important role in cerebral stroke and ischemia–reperfusion injury, further studies are required to determine the corresponding role of mitochondrial TET. These studies indicate that TET and 5hmC participate in multiple pathways to regulate brain tissue damage after cerebral stroke, suggesting a potentially effective therapeutic method for remyelination and neurological function recovery after cerebral infarction.

### 5.2 TET and Immune System Diseases

#### 5.2.1 TET and Autoimmune Diseases

*TET* expression and 5hmC contents play important roles in mediating adaptive immune functions involving immune cells and cytokines. More effort is currently being invested in understanding how TET proteins may influence the regulation of autoinflammatory and autoimmune diseases, characterized by immune tolerance disorders affecting specific tissues or the entire body in response to self‐antigens [134, 135]. Tanaka et al. [136] suggested that TET2‐ and TET3‐mediated DNA demethylation participates in the inhibition of CD86 in self‐reactive B cells, thus providing a novel mechanism for preventing autoimmunity. Teghanemt et al. [137] summarized the crucial role of TET in promoting regulatory T cells (Treg cells) differentiation and development, which are important adaptive cells involved in maintaining tolerance and preventing autoimmunity. Inflammasome‐related genes are also more likely to undergo demethylation modifications via the regulation of *TET2* in monogenic autoinflammatory diseases [138].

Vogt–Koyanagi–Harada (VKH) disease is a systemic autoimmune disease characterized by an attack of CD4^+^ T cells on melanocyte‐associated antigens. Zhang et al. [139] reported the demethylation of a leucine‐rich repeat containing 39 (LRRC39) to determine the frequency of T helper 1 (Th1) and Treg cells in a TET2‐dependent manner. They further identified interferon (IFN) regulatory factor 7 and DEAD/DEAH box helicase 11 as potential targets regulated by *TET* expression, providing potential therapeutic targets for VKH [139]. Pandey et al. [140] proposed that *TET2* deficiency in hematopoietic cells, especially T cells, is involved in spontaneous hepatic pathology in mouse models of autoimmune hepatitis (AIH). Defective *TET2* expression might affect the recruitment of IFN‐γ‐secreted CD8^+^ T cells and Thq cells, leading to severe autoimmunity [140]. However, the role of the TET/forkhead box p3 (FOXP3) pathway in regulating the abundance and activity in AIH is still unclear. Functionally impaired Treg cells accumulated and detected in the livers of patients with AIH were previously regarded as a result of the stimulation of cytokines secreted from monocytes [141, 142]. The involvement of DNA demethylation in this process requires further investigation. In addition, a possible relationship between *TET* expression and rheumatoid arthritis (RA) has been proposed. Kawabe et al. [143] identified the upregulation of *TET3* and 5hmC in the synovitis tissues of patients with RA. Tumor necrosis factor α (TNF‐α) seemed to be involved in the stimulation of TET3 and 5hmC levels in fibroblast‐like synoviocytes, leading to enhanced C‐X‐C motif ligand 8 and C‐C motif ligand 2 [143, 144]. Abnormal *TET2* expression in myeloid cells from the synovium in RA can be suppressed by glucocorticoids [145]. Moreover, in mice with multiple sclerosis and experimental autoimmune encephalomyelitis, decreased TET1, TET2, and 5hmC levels were associated with myelin damage [146]. Besides, the levels of TET2 and 5hmC were markedly enriched in epithelial cells, while the contents were decreased in inflammatory cells accumulated in labial salivary glands from patients with Sjögren's syndrome, which were induced by cytokines such as TNF‐α and IFN‐γ [147, 148].

Type 1 diabetes (T1D) is a β cellular autoimmune disease. Genetic and epigenetic modifications have crucial effects on the pathogenesis and development of T1D, suggesting potential therapeutic targets [149]. High levels of TET2 in T1D are closely associated with the regulation of miRNA. Stefan‐Lifshitz et al. [150] reported the crosstalk between IFN‐α, miR‐26a, and TET2 based on T1D mice models, indicating a TET‐dependent DNA demethylation induced by IFN‐α. Scherm et al. [151] demonstrated that miR‐142‐3p degraded TET2 in Treg cells, thereby interfering the Treg cells’ induction and stability. Improving *FOXP3* demethylation in Treg cells has been proven to be beneficial in preventing T1D in animal models and restoring a balanced state of diverse T cell subsets [152]. Furthermore, it has been proposed that TET2 controls the responses of pancreatic β cells to autoinflammation in T1D. According to Rui et al. [153], *TET2* expression was induced during β cell demises, while the opposite phenomena were observed in the survival groups. *TET* knockdown in β cells protects them from IFN‐γ‐induced inflammatory responses and the upregulation of pathologic genes [153].

Systemic lupus erythematosus (SLE) is a typical autoimmune disorder characterized by a specific profile of antinuclear antibodies that affect multiple systems, including the skin, kidneys, joints, heart, and nervous system [154, 155]. Alterations in the key regulatory genes of both innate and adaptive immune cells are considered the main pathological factors [156, 157]. Recent studies have highlighted the importance of chromatin methylation and demethylation, with a focus on investigating the indispensable role of TET proteins [136, 158]. A previous study identified a global hypomethylation pattern of several autoimmunity‐associated genes in T cells, B cells, and monocytes from the peripheral blood of patients with SLE [159, 160, 161]. Based on peripheral blood samples from patients with SLE, Luo et al. [162] reported that TET2 recruited by STAT pathways promoted DNA demethylation of the IFN‐inducible 44‐like (IFI44L) promoter, which induced the overexpression of IFI44L in monocytes, leading to the secretion of proinflammatory cytokines and the activation of monocyte‐derived dendritic cells. In Treg cells from patients with lupus, elevated TET2 is involved in the expression of FOXP3, leading to an increased frequency of CD4^+^FOXP3^+^T cells in the circulation as well as in renal biopsy specimens [163, 164]. In inflammasomes from patients with SLE, accumulation of TET2 was also observed in the absent in melanoma 2 promoter, leading to the differentiation of memory B cells and plasma T follicular helper (TFH) cells within the lesions [165, 166]. In addition, Sung et al. [167] revealed a potential correlation between serum levels of antidouble‐stranded DNA antibody and complement concentrations (C3 and C4) and the expression of *TET2*, suggesting that TET may play an important role. Currently, it can be concluded that epigenetic modifications are closely related to the occurrence, progression, treatment, and activity of SLE; however, there are still many unknowns regarding TET‐mediated DNA demethylation. In addition, numerous possible mechanisms lead to DNA demethylation in patients with SLE, including oxidative stress (OS), regulation of noncoding RNA, and iron metabolism [168, 169, 170]. Therefore, we need to further explore whether aberrant *TET* expression is involved in the mechanisms that collectively contribute to low methylation in SLE.

#### 5.2.2 TET and Allergy

Allergy refers to an allergen‐specific immunoglobulin E‐mediated hypersensitive reaction. Epigenetic modifications are at the intersection of environmental exposure and cellular responses, among which DNA methylation and demethylation at CpG have been extensively studied [171, 172].

Allergic bronchial asthma is the most common type of asthma and is characterized by eosinophil recruitment, excessive mucus secretion, high responsiveness of the trachea, and reversible tracheal obstruction [173]. The expression of *TET* may show a pattern of up‐ or downregulation in different cells as well as in allergic diseases, depending on the cell type involved and its specific role in the immune response. In children with allergic asthma, the methylation levels of the *TET1* promoter region decrease, resulting in an increase in *TET1* expression in bronchial epithelial cells, followed by an increase in the global 5hmC contents [174]. Additionally, in patients with bronchial asthma, the expression of *TET2* in Treg cells decreases, leading to an increase in the methylation of the FOXP3 promoter region and a decrease in FOXP3 expression, which contributed to the loss of their ability to maintain homeostasis and regulate inflammatory responses [175, 176, 177]. In a mouse model of asthma, Yeung et al. [178] observed that the expression levels of *TET1* and isocitrate dehydrogenase (IDH), as well as the levels of 5hmC and α‐KG, were significantly elevated in airway smooth muscle cells, and these changes further triggered the overexpression of transforming growth factor‐β2 and increased cell proliferation. Mechanistic studies showed that upregulation of IDH gene expression and accumulation of its catalytic product α‐KG, by enhancing TET activity triggers DNA hydroxymethylation, which ultimately drives the transformation of airway smooth muscle cells to a profibrotic, hyperproliferative, and aberrant phenotype [178].

In allergic rhinitis mouse models, a lower abundance of 5hmC was observed in the nasal mucosa, and allergic rhinitis mice *TET2* knockout developed severe allergic responses [179]. Meng et al. [179] further confirmed that the loss of TET2 induces overexpression of major histocompatibility complex molecules, accompanied by an altered proinflammatory cytokine profile. Defective TET2 is also associated with FOXP3 DNA methylation, leading to a decrease in Treg cell percentage [180]. In a previous study focusing on chronic hypersensitivity pneumonia, reduced TET2 levels were observed in the lung tissues of patients with chronic hypersensitivity pneumonia, and is considered the primary regulatory factor for 5mC and 5hmC regulation [181]. Nucleotide‐binding and oligomerization domain‐like receptors and Forkhead box O pathways might be involved in the aggregation of Th1 cells and eosinophils in diseased lung tissue due to aberrant demethylated modifications [181]. In conclusion, these studies provide new insights into the potential role of TET‐dependent DNA demethylated marks in response to allergen challenges, suggesting promisingly utilized as biomarkers for the diagnosis, treatment, and prognosis of the allergy.

### 5.3 TET and Metabolic Diseases

#### 5.3.1 TET and Diabetes

Diabetes is a group of metabolic disorders characterized by impaired glucose utilization and excessive production due to abnormal gluconeogenesis and glycogenolysis, leading to hyperglycemia [182]. High glucose levels lead to global DNA hypomethylation and aberrant gene expression. Even when normal blood glucose levels are restored through medical intervention, the high glucose‐induced cytosine hypomethylation across the genome during diabetes cannot be reversed [183]. In 2019, Yuan et al. [184] collected and detected TET, 5mC, and 5hmC in blood samples from humans and rats with diabetes. These results suggest that lower 5mC and higher 5hmC levels are risk factors for diabetes and may be regulated by elevated levels of TET2 and TET3. Whereas targeted inhibition of both TET2 and TET3 helps to restore glucose homeostasis [185, 186].

Throwing light on various factors contributing to the pathogenesis and progression of diabetes, epigenetic modifications, especially the imbalanced states between DNA methylation and demethylation have been highlighted owing to their potent correlation with the expression of related genes involved in insulin, hyperglycemia, and diabetesx [187]. Abnormal DNA demethylation levels have been recognized as both the cause and result of diabetes [188]. Emerging evidence demonstrates the pivotal involvement of TET and DNA demethylation in the pathogenesis of diabetes, partly attributed to errors in transcriptional responses, especially evident during the aging process. For example, the transformation of white adipocytes into brown/beige adipocytes in response to excessive fatty acids declines during aging, leading to metabolic dysfunction, primarily obesity and diabetes. Tian et al. [189] have proposed that a decrease in the enzymatic capacity of TET in aged mice, leading to a lack of DNA demethylation in the Prdm16 promoter required for brown/beige adipogenesis, is a potential factor contributing to the development of aging‐related impaired glucose and lipid tolerance. In addition, TET proteins in adipocytes function as inhibitors of β‐adrenergic receptor signaling. Increased TET‐dependent DNA demethylation leads to lower responsiveness to the required energy expenditure, inducing insulin resistance [190]. Recently, Xie et al. [191] proposed that TET3 overexpressed in agouti‐related peptide‐expressing neurons located in the hypothalamic activity‐regulated cytoskeletal‐associated functions as a crucial downstream regulator, epigenetically defecting leptin‐mediated pathways and further causing hyperphagia, obesity, and diabetes.

The impact of hyperglycemia on TET‐mediated DNA demethylation can be divided into two possibilities: first, altered cofactors under diabetic conditions may regulate the expression and activity of TET protein directly, and second, key enzymes involved in TET‐mediated DNA demethylation are strongly affected. As mentioned previously, tricarboxylic acid (TCA) cycle metabolites mediate the degree of 5hmC and the demethylation of target genes by regulating TETs’ activity and content [192]. Primarily, the growing abundance of glutamine, glutamate, and α‐KG in diabetics strongly elevated TET levels; additionally, expression of *TET* may be inhibited by the downstream TCA metabolites, especially succinate [193]. Besides, diabetic conditions cause a decrease in serum ascorbic acid concentration, inhibiting TET‐mediated 5mC oxidation and introducing a high methylation state [194, 195]. Hyperglycemia also affects TET‐dependent DNA demethylation activities by mediating the expression of regulatory enzymes at each demethylation step, including Gadd45a, TDG, and PARP [196]. Intervention with targeted inhibitors of these enzymes is beneficial for ameliorating diabetes‐related complications such as peripheral neuropathy, cardiovascular disorders, and glomerular fibrosis [197, 198, 199]. Moreover, several studies have proposed a third possibility for altered *TET* expression in patients with diabetes. Given the elevated OS in patients with diabetes, excessive OS is regarded as an important pathogenic factor contributing to numerous complications associated with diabetes [200, 201]. Previous genome‐wide profiling data indicated a global decrease in DNA demethylation under oxidative conditions, whereas several specific DNA sites with high levels of 5hmC were possibly attributed to antioxidant gene expression regulated by the recruitment of TET [202]. This suggests that OS is greatly involved in regulating the expression of *TET* during the progression of diabetes and that TET‐dependent DNA demethylation may also affect the content of intracellular reactive oxygen species to regulate the cellular damage caused by hyperglycemia.

Diabetes is also a susceptibility factor for a range of long‐term chronic disorders, such as microvascular diseases, cardiomyopathy, neuropathy, and chronic kidney disease [203]. Several complications implicated in the development of diabetes are affected by altered DNA methylation, and we have summarized the aberrant levels of TET‐dependent DNA demethylation under these conditions based on the latest developments [204].

Abnormal levels of TET‐induced DNA demethylation have been reported to be responsible for altered expression of genes related to endothelial dysfunction and further cardiovascular disease caused by diabetes [205]. In 2018, Zhao et al. [206] analyzed *TET* expression in endothelial progenitor cells and found that the mRNA levels of *TET1* increased, whereas those of *TET2* and *TET3* decreased in patients with diabetes and peripheral artery disease. They further demonstrated decreased levels of *TET3* mRNA and TET3 protein in endothelial progenitor cells as biomarkers to evaluate angiopathy in patients with diabetes. Regarding retinal arterioles, hyperglycemia triggers the overexpression of *TET2*, which is responsible for the demethylation of the endothelial cell‐specific factor roundabout 4, leading to binding with specificity protein 1, accelerating retinal vascular leakage, and abnormal angiogenesis in the progression of diabetic retinopathy [207]. In addition, based on data from retinal samples of diabetic mice, altered TET levels are involved in the activation of MMP‐9 expression, which has been identified as a trigger of retinal mitochondrial injuries and diabetic retinopathy [208]. Decreased levels of TET2 have been detected in the cerebral cortex of diabetic mice, and the subsequent decrease in 5hmC levels was involved in neuronal apoptosis [209, 210]. In contrast, the activation of the mitochondrial TCA cycle and AMP‐activated kinase (AMPK) pathways helps maintain TET‐dependent DNA demethylation, alleviates injuries to the brain, and fulfills neuroprotective activity in diabetes [210]. Previous studies have shown a higher risk of cancer in patients with diabetes [211, 212].

Mechanistically, elevated blood glucose levels impede AMPK‐mediated phosphorylation of TET proteins, which leads to destabilization of the tumor suppressor TET2 and a decrease in 5hmC levels [213]. Moreover, decreased 5hmC levels are an epigenetic marker of cancer. This also explains why the antidiabetic drug metformin exerts a tumor‐suppressive effect from an epigenetic perspective. Furthermore, hyperglycemia contributes to the O‐GlcNAcylation of TET1 via linkage with GlcNAc, acting as a crucial trans‐acting element during feed‐forward regulation and subsequently enhancing the production of O‐GlcNAc transferase. Overactivation of this pathway increases the risk of triple‐negative breast cancer (TNBC), particularly in menopausal women [214]. Aiming at hepatocellular carcinoma (HCC), the interactomic approach between nuclear receptor REV–ERBα (nuclear receptor subfamily 1 group D member 1) and O‐GlcNAc transferase participates in ensuring the rise in contents and O‐GlcNAcylation of TET [215]. Decreased expression of *TET1* and *TET3* in the glomerulus has been observed in female mice with diabetic kidney disease, whereas *TET2* expression was found to be elevated in male rats [216, 217]. Yang et al. [217] suggested that overactivated TET2 accumulated in and further bound with the TGFβ1 regulation region, demonstrating fibrotic levels and cell phenotype transformation. Tampe et al. [218, 219] revealed that impaired TET3 results in the hypermethylation of genes related to the activation of fibroblasts, promoting kidney fibrosis. In diabetic cardiomyopathy, the notable accumulation of 5mC and 5hmC regulated by TET family members has been investigated, illustrating the possible role engaged in metabolic pathways and hyperglycemic damage [220, 221]. In both diabetic cardiac mesenchymal cells (CMSCs) and the whole heart of diabetic mice, defects TET1 nuclear localization have been observed, while administration of α‐KG helps to restore DNA methylation and improve insulin sensitivity [198]. Compared with patients with nondiabetic wounds, those with diabetic foot ulcers showed an altered expression pattern, characterized by elevated α‐KG, TET2, and MMP‐9. Upregulated TET2‐induced site‐specific DNA demethylation plays a vital role in MMP‐9 expression and is a biomarker for predicting poor wound healing [222].

Gestational diabetes mellitus (GDM) is a common clinical condition in pregnancy that results in adverse clinical outcomes for both the mother and the fetus, especially impaired development of the fetal vascular systems [223]. Recent studies on genetics and epigenetics have highlighted the novel underlying mechanisms of GDM pathophysiology, suggesting feasible approaches to identify potential risks and therapeutic interventions [224]. Sun et al. [225] illustrated insufficient expression of *TET2* in the umbilical veins of GDM, and the lowered 5hmC abundance subsequently exerts a negative effect on vital biological roles, leading to adverse pregnancy outcomes. Moreover, poor maternal diet or maternal obesity is highly correlated with increased vulnerability to metabolic disorders, including type 2 diabetes, in offspring, which could be partly attributed to the suppression of AMPK/TET signaling pathways in fetal livers [226]. Loss of demethylation at the promoters of glucose metabolism genes impairs glycoregulatory activity in hepatocytes, which fails to improve glucose homeostasis [226].

Therefore, in the case of glucose metabolism disorders and diabetes, TET‐mediated DNA demethylation is regulated by several factors, and completely contradictory effects have already been observed, which may partly explain the conflicting results regarding *TET* expression in different clinical trials and animal models. Future studies must be conducted with caution before their application to clinical interventions.

#### 5.3.2 TET and Nonalcoholic Fatty Liver Disease

Nonalcoholic fatty liver disease (NAFLD) has a high incidence rate, especially in Western countries, and can develop into end‐stage liver diseases, including cirrhosis and HCC. Current data obtained from animal models and human liver tissues indicate that abnormal DNA methylation at particular sites, including genes related to energy metabolism, is an important pathogenic mechanism of NAFLD [227, 228].

In 2018, Lyall et al. [229] proposed a novel modeling method to precisely preserve the content of 5hmC and TET proteins in NAFLD based on human hepatocyte‐like cells and the administration of lactate, pyruvate, and octanoic acid. Accumulated lipids in hepatocytes can be rescued by the overexpression of *TET1* and the cofactor for TET families [230]. In chronic liver disease, especially liver fibrosis, an increase in the expression of three DNMTs, DNMT1, DNMT3A, and DNMT3B, and a decrease in TET protein levels have been observed [231]. Abnormal levels of both DNMTs and TETs are responsible for the hypermethylation and demethylation of diverse genomic alterations, which are manifested in promoting low methylation and overexpression of pathogenic genes in the early stages of NAFLD, and in a later stage, the hypermethylation and silencing of genes that resist disease progression form a second strike to hepatocytes [232]. Highly expressed in liver tissues, the nuclear peroxisome proliferator‐activated receptors (PPARs) function as the dominant regulators of lipid metabolism in hepatocytes [233]. A previous observational study conducted by Pirola et al. [234] showed that genetic variations observed in TET1 and TET2 were highly correlated with abnormal PPARs methylation and hepatocyte injury, leading to the development of NAFLD. They further proposed that insufficient TET‐mediated demethylation levels mainly led to a decrease in the 5hmC content of mitochondrial DNA rather than in the nuclear genome [234]. In patients with NAFLD, specific CpG dinucleotides within the human PPARα and PPARγ gene promoters were silenced by DNA hypermethylation, which also seemed to be correlated with the severity of NAFLD [235]. However, acontroversy arose when Lee et al. [236] reported the involvement of TET2 in the demethylation and overexpression of c‐Maf‐inducing protein, which further induced the upregulation of guanylate binding protein 2/PPARγ/CD36 axis, accelerating the progression of NAFLD.

Considering that the decrease in genomic methylation levels in NAFLD may depend on the lack of methyl group donors in high‐fat dietary patterns, there is still a lack of evidence to explain the association between changes in *TET* expression and its mediated DNA demethylation with characteristic genes involved in the pathogenesis and progression of NAFLD [237]. However, abnormal methylation levels of certain primary metabolic genes in hepatocytes, such as apolipoproteins, fail to return to normal levels by improving diet and other approaches, indicating the possibility of other factors involved in DNA hypomethylation, with TET as a potential regulator [238]. Moreover, such abnormal methylation levels seem to be inherited by the next generation, leading to susceptibility to NAFLD or other metabolic disorders in the offspring [239, 240]. Therefore, it is important to identify the role of TET at specific hypomethylation sites.

### 5.4 TET and Cancer Development

Cancer cells exhibit highly dysregulated DNA methylation profiles, which can lead to genomic instability and aberrant expression of tumor suppressor genes or oncogenes [241]. Over the past few decades, dysregulation of cellular methylation landscapes has generated considerable interest in the field of cancer epigenetics. Disruption of dynamic DNA methylation programming has been observed in a variety of malignancies and has become a hallmark of some cancers [72]. However, this disruption of methylation programming showed remarkable heterogeneity in different tumor tissues, as evidenced by the upregulation of *TET* expression in some tumors and downregulation in others (Table 2).

**TABLE 2:** Differences in TET levels between tumor and normal tissues.

Cancer	Expression protein or mRNA	Number of samples	TET1/2/3 expression	Level of 5hmC	References
CRC	mRNA level	49	TET1↓, TET2↓, TET3–	↓	[242, 243]
	Protein level	19	TET1↓, TET2–, TET3–	NR	[242]
Pancreatic cancer	mRNA level	15	TET1↓, TET2–, TET3–	↓	[244]
	Protein level	63	TET1↓	NR	[244]
Gastric cancer	mRNA level	33/76	TET1↑, TET2↑, TET3↑	−	[245]
	Protein level	33	TET1–, TET2–, TET3–		[245]
	mRNA level	58	TET1↓, TET2↓, TET3↓	↓	[246]
Breast cancer	mRNA level	15	TET1↓, TET2↓, TET3↓	↓	[247]
Prostate cancer	Protein level	40	TET1↓	↓	[247]
Prostate cancer (Metastatic tumor)	mRNA level	19	TET2↓	NR	[248]
Ovarian cancer	mRNA level	53	TET1↑, TET2–, TET3–	↑	[249]
Lung cancer	mRNA level	50	TET1↑	NR	[247]
Hepatocellular carcinoma	mRNA level	108	TET1↓, TET2↓, TET3↓	↓	[250]
	Protein level	108	TET1↓, TET2↓, TET3↓		[250]
	mRNA level	49	TET1↓, TET2↓, TET3↓	↓	[247]
Cholangiocarcinoma	mRNA level	36	TET1↑, TET2↑, TET3↑	−	[251]
	Protein level	91	TET1↑, TET2↓, TET3↑		[251]
Glioblastoma	mRNA level	21	TET1↓, TET2↓, TET3↓	↓	[252]

– indicates no change in the level of expression; ↑ indicates an increase in the level of expression; and ↓ indicates a decrease in the level of expression.

Abbreviations: 5hmC, 5‐hydroxymethylcytosine; CRC, colorectal cancer; NR, not reported; TET, ten‐eleven translocation.

#### 5.4.1 TET and Solid Tumors

**FIGURE 4:**
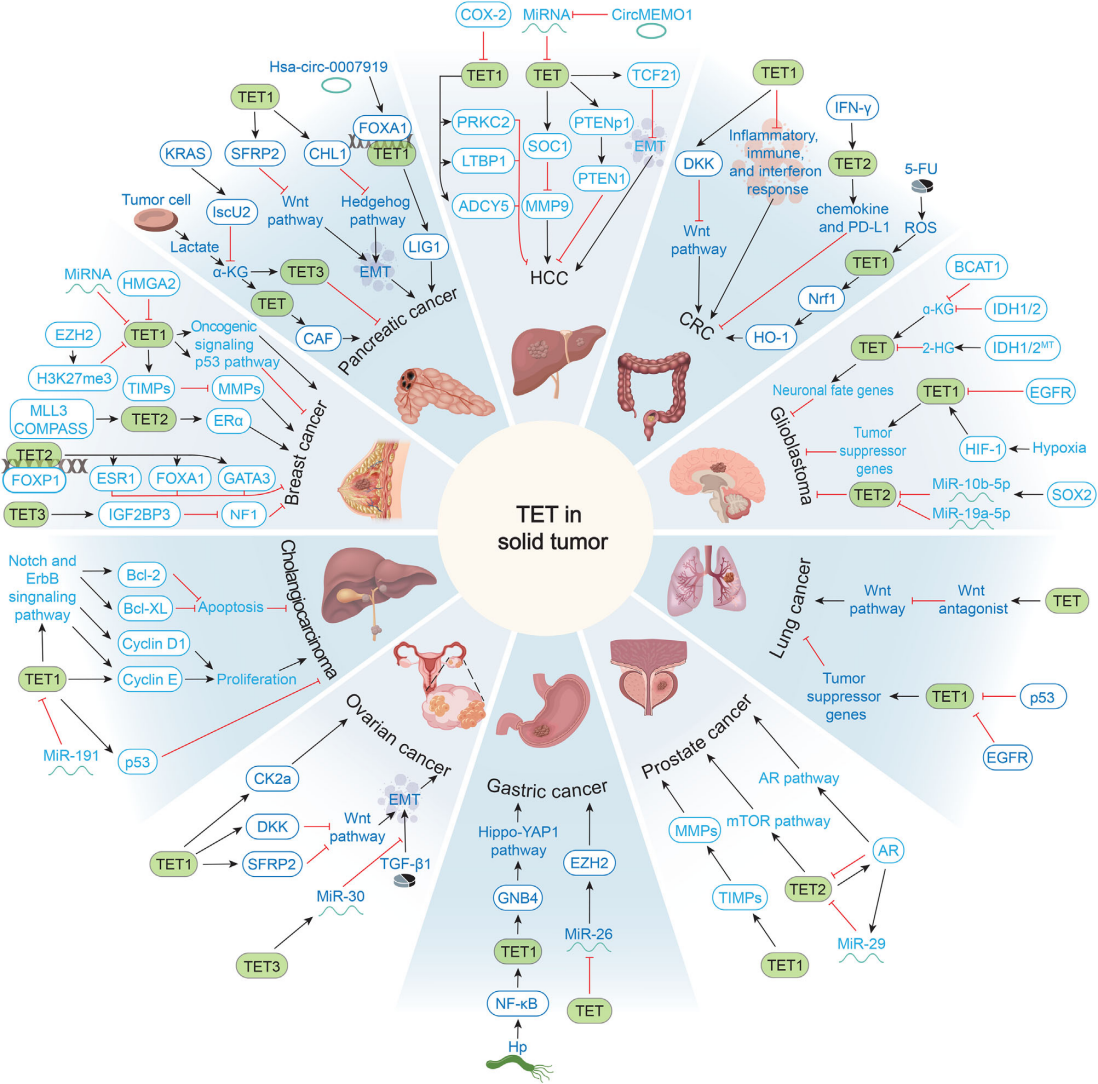
The role of TET proteins in cancer is heterogeneous. Dysregulation of TET involved in the development of a variety of cancers, including breast, pancreatic, lung, prostate, gastric, and ovarian cancers as well as cholangiocarcinoma, HCC, CRC, and glioblastomas. Abbreviations: TET, ten‐eleven translocation; HCC, hepatocellular carcinoma; CRC, colorectal cancer; MMP, matrix metalloproteinase; TIMPs, tissue inhibitors of metalloproteinases; ERα, estrogen receptor alpha; HMGA2, high mobility group AT‐hook 2; SFRP2, secreted frizzled‐related protein 2; EMT, epithelial–mesenchymal transition; α‐KG, α‐ketoglutarate; CAF, cancer‐associated fibroblast; DKK, Dikkopf; ROS, reactive oxygen species; 5‐FU, 5‐fluorouracil; 2‐HG, 2‐hydroxyglutarate; IDH, isocitrate dehydrogenase; HIF‐1, hypoxia‐inducible factor‐1; BCAT1, branched‐chain amino acid transaminase 1; AR, androgen receptor; Hp, helicobacter pylori; GNB4, guanine nucleotide‐binding protein subunit beta‐4; SFRP2, secreted Fzd receptor protein 2; CK2α, casein kinase II subunit alpha.

The mutation rate of *TET* in solid tumors is relatively low but is usually accompanied by changes in TET protein activity or expression [16, 253, 254]. The contribution of TET to oncogenesis in solid tumors remains largely unknown. The global decline in the TET catalytic product 5hmC was initially considered a hallmark of cancer, but it is now generally accepted that TET functions both as a tumor suppressor gene and an oncogene, indicating that there may be heterogeneity in the role of TET proteins in cancer pathogenesis (Table 2) [255]. This section focuses on the impact of TET1/2/3 activity and function on the development of various common solid cancers (Figure 4). These generalizations may resolve confounding reports and provide opportunities for novel therapeutic interventions.

##### 5.4.1.1 Breast Cancer

Breast cancer is the most common cancer and the leading cause of cancer‐related deaths in women [256]. In 2011, Haffner et al. [257] first reported that breast cancer tissues exhibit lower 5hmC levels than adjacent normal tissues. However, other studies have shown that the degree of *TET* expression, which catalyzes the generation of 5hmC, varies between breast cancer subtypes [258]. Additionally, some studies have reported TET1 as a tumor suppressor gene in breast cancer, but it also exhibits oncogenic properties in other breast cancer studies. This conflicting evidence raises the interesting possibility that TET1 is both an oncogene and a tumor suppressor [258, 259]. TNBC is the lowest‐methylated breast cancer subtype. Approximately 40% of patients with TNBC overexpress *TET1* compared with normal breast tissue, with TET1 exhibiting oncogenic properties [258]. Upregulated *TET1* acts as an oncogene, leading to the hypomethylation and activation of cancer‐specific oncogenic signaling pathways (including phosphoinositide 3‐kinase [PI3K], epidermal growth factor receptor [EGFR], and platelet‐derived growth factor), which results in the development of TNBC [258, 260]. However, patients with low TET1 and high enhancer of zeste homolog 2 (EZH2) exhibit the worst survival outcomes [261]. This finding was supported by the fact that, in TNBC cells, EZH2 reduces *TET1* expression through H3K27me3 epigenetic regulation and subsequently inhibits the antitumor p53 signaling pathway [261]. Moreover, TET1 inhibits tumor cell invasion by maintaining the expression of tissue inhibitors of metalloproteinases (TIMP) and MMP [262]. In hormone receptor positive breast cancers (HRBC) and human EGFR 2‐like tumors, TET1 was found to be significantly downregulated [258, 263]. The mechanism of its downregulation involves the negative regulatory effects of a series of molecules on TET1, including high mobility group AT‐hook 2, miR‐29a, and miR‐22 [264, 265, 266]. In these studies, the switch between carcinogenic and anticancer properties of TET1 highlights the complex network of relationships between TET1 and breast cancer.

In contrast, *TET2* expression is downregulated in both HRBC and TNBC [258]. Estrogen receptor (ER) signaling is a key factor in the development of hormone receptor‐positive (HR^+^) breast cancer [267]. Based on this characterization, ER signaling can be downregulated by antiestrogen therapy to inhibit the development of HR^+^ breast cancer [267, 268]. ERα is a major nuclear factor mediating estrogen signaling [269]. However, deletion of *TET2* affects the recruitment of ERα to active enhancers, which disrupts the mixed‐lineage leukemia 3 COMPASS–TET2–ERα axis and attenuates the estrogen response, leading to the development of endocrine therapy resistance [270, 271]. In addition, TET2 forms a chromatin complex with forkhead box protein 1, which mediates the demethylation of three key genes, estrogen‐receptor 1, GATA binding protein 3, and Forkhead box protein A1 (FOXA1), that regulate luminal lineage commitment and endocrine responses [271]. *TET2* deletion leads to methylation silencing of these genes and promotes resistance to antiestrogen therapy [271]. *TET3* is upregulated in both HRBC and TNBC [258]. TET3‐mediated hypomethylation of the insulin‐like growth factor 2 mRNA‐binding protein 3 (IGF2BP3) promoter and upregulation of IGF2BP3 expression decrease neurofibromatosis type 1 stability, leading to the development of TNBC [272]. Overall, the current understanding suggests that the involvement of TET in breast cancer stems from the intertwining of multiple signaling pathways. The exact TET protein or signaling pathway that mediates its primary role in different breast cancer subtypes needs to be further explored.

##### 5.4.1.2 Pancreatic Cancer

Pancreatic cancer is one of the deadliest malignant tumors, with a 5‐year survival rate of merely 10% for affected patients [273]. Aberrant DNA methylation in pancreatic cancer has been extensively studied [274, 275]. In pancreatic cancer, TET1 levels and 5hmC content are downregulated, low levels of TET1 are associated with short overall survival, and TET1 acts as a tumor suppressor [244, 276]. Molecularly, downregulated TET1 reduces demethylation of the promoter of secreted frizzled‐related protein 2 (SFRP2), an inhibitor of the Wnt/β‐catenin pathway, which represses SFRP2 transcription, activates both classical and nonclassical Wnt signaling pathways, and ultimately leads to epithelial–mesenchymal transition (EMT) in pancreatic cancer [244]. Aberrant activation of EMT promotes cancer metastasis, increases tumor stemness, and enhances resistance to chemotherapy and immunotherapy [277]. Additionally, TET promotes tumor cell invasion and metastasis by affecting the formation of cancer‐associated fibroblasts (CAF) [278]. Lactate production by tumor cells leads to increased production of α‐KG in mesenchymal stem cells (MSCs), which activates the TET enzyme, leading to an increase in hydroxymethylation and promoting the differentiation of MSCs into CAFs [278]. In pancreatic ductal adenocarcinoma (PDAC), activated kirsten rat sarcoma viral oncogene homolog (KRAS) upregulates iron‐sulfur clusters 2 expression and promotes α‐KG catabolism in mice, which leads to suppression of TET3 and DNA 5mC‐dependent PDAC cell proliferation [279]. In addition, TET1 is involved in the development of drug resistance in pancreatic cancer. TET1 promotes close homologue of L1 (CHL1) transcription by binding to and demethylating the CHL1 promoter, thereby inhibiting the Hedgehog pathway [276]. Activation of the Hedgehog signaling pathway is associated with EMT and drug resistance in pancreatic cancer. Downregulation of *TET1* results in the activation of the Hedgehog signaling pathway, promoting EMT and rendering PDAC cells insensitive to 5‐fluorouracil (5‐FU) and gemcitabine [276]. TET1 is also involved in other chemotherapy resistance mechanisms. Xu et al. [280] found that gemcitabine‐resistant PDAC tissues and cells express high levels of hsa‐circ‐0007919, which recruits FOXA1 and TET1 to reduce DNA ligase 1 (LIG1) promoter methylation and enhance LIG1 transcription. LIG1 is involved in multiple DNA repair pathways that reduce chemotherapy‐mediated DNA damage, leading to chemotherapy resistance [280].

##### 5.4.1.3 Hepatocellular Carcinoma

The functions of TET family members in HCC are of great interest, with several studies demonstrating the marked downregulation of the *TET1/2/3* expression in HCC [247, 250, 281‐283]. Consistently, the level of the TET catalytic product 5hmC is also downregulated in HCC tissues and cell lines [284]. Of the three TETs, TET1 has received the most attention. Chen et al. [285] showed that the upregulation of cyclooxygenase‐2 in HCC promotes HCC formation by decreasing the expression of *TET1*, which in turn reduces the expression of the tumor suppressor genes TGFβ‐binding protein 1, adenylyl cyclase 5, and protein kinase C zeta. Additionally, TET1 is regulated by several miRNAs [250, 286‐288]. Chen et al. [250] showed that miR‐29a overexpression in HCC silenced cytokine signaling 1 by negatively regulating *TET* expression, which triggered the upregulation of MMP‐9 and increased tumor invasiveness and metastasis. Cao et al. [289] proposed that exosomal miR‐21 negatively regulates phosphatase and tensin homolog (PTEN) and PTENp1 expression by decreasing *TET* expression, which promotes HCC development. The miRNAs that regulate *TET* are also regulated by circular RNAs [290]. CircMEMO1 regulates TET levels by sponging miR‐106b‐5p, which promotes transcription factor 21 expression and inhibits EMT and HCC development [290]. Compared with TET1, the mechanisms of action of TET2 and TET3 in HCC have been poorly studied. Unlike most studies, Sajadian et al. [291] proposed that the reduction in 5hmC in HCC is mainly attributed to the impaired expression and activity of TET2 and TET3, rather than that of TET1. In addition, this study found that the anticancer drug 5‐azacytidine (5‐AZA) prevents HCC tumor cell growth by inducing the expression of *TET2* and *TET3* [291]. These studies demonstrate the importance of TET in the development and progression of HCC.

##### 5.4.1.4 Colorectal Cancer

Colorectal cancer (CRC) is the third most common cause of cancer‐related deaths worldwide [292]. TET proteins usually act as tumor suppressors in CRC. The levels of TET1/2 and 5hmC were markedly lower in patients with CRC than in normal colonic tissue [242, 243]. The mechanism by which downregulation of *TET* promotes CRC progression has been partially elucidated. Deletion of *TET1* and TDG not only leads to global hypermethylation of DNA in CRC, especially CpG islands, but also leads to the upregulation of genes related to inflammation, IFN, and immune responses, which may be associated with the malignant transformation of tumors and immune escape [293]. Wnt signaling is critical for angiogenesis and tumor growth in CRCs, and most CRCs are triggered by aberrant activation of the Wnt/β‐catenin signaling pathway [294, 295]. TET1 binds to the promoter of the Dikkopf (DKK) gene, an inhibitor of Wnt signaling, to maintain its hypomethylation, thereby inhibiting Wnt signaling [296]. When TET1 is downregulated, the Wnt signaling pathway is activated, promoting the development of CRC. In addition, Xu et al. [254] showed that the IFN‐γ/JAK/STAT/TET pathway controls the expression of chemokines and PD‐L1, lymphocyte infiltration, and cancer immunity. Deletion of *TET2* in colon tumor cells reduces chemokine and PD‐L1 expression, as well as tumor lymphocyte infiltration, allowing tumors to evade antitumor immunity and resist anti‐PD‐L1 therapy [254]. Notably, TET, as a tumor suppressor gene, is involved in the development of fluoropyrimidine 5‐FU resistance [297]. In 5‐FU‐resistant cells, 5‐FU‐generated reactive oxygen species upregulates the expression and function of *TET1*, which leads to the upregulation of nuclear factor‐erythroid 2‐related factor 2 and heme oxygenase‐1 expression and the development of resistance to chemotherapeutic agents in cancer cells [297].

##### 5.4.1.5 Glioblastoma

Glioblastoma accounts for approximately half of all malignant brain tumors, with a median survival of less than 2 years [298]. It has been shown that *TET1/2/3* expression is downregulated in glioblastoma and that TET3 levels are associated with a better prognosis in patients with glioblastoma [252, 299, 300]. Consistently, glioblastomas show markedly less abundance of 5hmC than normal brain regions, and there is a strong relationship between low levels of 5hmC and reduced survival in glioblastomas [301, 302]. These observations are partly explained by the high frequency of IDH1/2 mutations in glioblastoma, which result in the production of 2‐hydroxyglutarate (2‐HG) (α‐KG correspondingly reduced) that inhibits TET enzyme function [303, 304]. This is similar to the inhibition of TET activity by *IDH1/2* mutations in hematological cancers. Reduced TET activity through α‐KG depletion, has also been observed in branched‐chain amino acid transaminase 1 (BCAT1) [305]. Boskovic et al. [305] found that BCAT1 in glioblastoma promotes tumor growth through α‐KG depletion leading to reduced TET activity and inhibition of differentiation‐related genes. In addition, oncogenic EGFR induces the silencing of tumor suppressor genes by inhibiting TET1 and promoting glioblastoma development [306]. Notably, TET1 participates in the regulation of hypoxia and promotes cancer development [258, 307]. Hypoxia‐inducible factor‐1 (HIF‐1) is essential for glioblastoma development; hypoxia leads to the upregulation of HIF‐1, which promotes the transcriptional activation of *TET1* and the induction of hypoxia‐responsive genes [307, 308, 309]. For TET2, Lopez‐Bertoni et al. [300] found that the reprogramming transcription factor SOX2, which is highly expressed in glioblastoma, leads to reduced TET2 levels through the activation of miR‐10b‐5p. *TET2* deletion enhances stemness in glioblastoma cells and induces a more aggressive tumor phenotype [300]. Similarly, miRNAs involved in *TET2* regulation to mediate glioblastoma development also include miR‐19a‐5p [310]. Taken together, these studies suggest that TET plays a unique role in glioblastoma genesis and development.

##### 5.4.1.6 Lung Cancer

Lung cancer is a major cause of cancer‐related death [311]. The loss‐of‐function mutations in *TET* are found in 7.4% of human lung adenocarcinomas, with *TET1*, *TET2*, and *TET3* mutations accounting for 4, 1.6, and 1.8%, respectively [253]. Consistently, TET‐mediated generation of 5hmC is considerably reduced in lung cancer [312]. Impairment of these TET functions reprograms the DNA methylation landscape and silences the expression of Wnt antagonists, leading to the hyperactivation of Wnt signaling and enhanced tumorigenic potential [253]. Notably, mutations in TET frequently occur in conjunction with oncogenic *KRAS* mutations, prompting a focus on the interactions between TET and other oncogenes [253]. However, the role of TET1 in lung cancer remains unclear. Filipczak et al. [313] showed the overexpression of *TET1* in adenocarcinoma and squamous cell carcinoma, functioning as an oncogene in lung cancer. Mechanistically, the transcription of TET1 is negatively regulated by p53, and mutations in p53 lead to *TET1* overexpression in lung cancer and promote tumor cell proliferation and growth [313]. In contrast, other studies have defined TET1 as a cancer suppressor [306, 314]. Forloni et al. [306] proposed that EGFR induces the silencing of tumor suppressor genes through the inhibition of TET1 in lung cancer, thereby promoting cancer development. These opposing findings may account for the heterogeneity of TET in different lung cancer subtypes and tumor microenvironments.

##### 5.4.1.7 Prostate Cancer

Widespread dysregulation of the epigenetic landscape in prostate cancer is a hallmark of tumorigenesis [315, 316, 317]. Studies have shown a reduction in TET1/2 and 5hmC levels in prostate cancer [247, 248, 318]. Consistent with the oncogenic mechanisms mentioned earlier in breast cancer, *TET1* also inhibits tumor cell invasion by activating TIMPs, whose depletion promotes prostate cancer invasion and metastasis [262]. In contrast, TET2 plays a unique role in prostate cancer. Androgens and androgen receptors (AR) play key roles in prostate cancer development, and TET2 physically interacts with AR and its coactivators to affect AR signaling [248, 319]. In turn, TET2 is inhibited by AR in prostate cancer cells through two pathways: AR‐induced inhibition of *TET2* expression by the miR‐29 family, and direct binding of AR to the enhancer region of *TET2* and inhibition of its transcription [320]. TET2 inhibition and decreased 5hmC levels activate key prostate cancer‐related pathways, including the mechanistic target of rapamycin kinase (mTOR) and AR pathways, which drive prostate cancer development [320]. These studies highlight the complexity of the regulatory role of TET in prostate cancer.

##### 5.4.1.8 Gastric Cancer

Gastric cancer is the fifth most common cancer worldwide [311]. TET1/2/3 transcript levels are upregulated in gastric cancer and are involved in gastric cancer development by interacting with multiple molecules [245, 321]. *Helicobacter pylori* (Hp) infection is a risk factor for gastric cancer development [322, 323]. Liu et al. [321] showed that Hp infection activates NF‐κB, which upregulates the expression of *TET1*. TET1 binds to the guanine nucleotide‐binding protein subunit beta‐4 (GNB4) promoter region, which promotes gastric cancer proliferation and metastasis by activating the transcription of GNB4 and activation of the Hippo‐Yes‐associated protein 1 pathway [321]. Additionally, TET proteins interact with miRNAs through noncatalytic functions to promote gastric cancer development. MiR‐26 is a functional miRNA with oncogenic effects, and the 3′UTR of the TET is capable of forming a complex with miR‐26, which reduces the inhibitory effect of miR‐26 on the target gene EZH2 and promotes gastric carcinogenesis [245].

##### 5.4.1.9 Ovarian Cancer

Ovarian cancer is the most common gynecologic malignancy [324]. However, the role of TET1 in ovarian cancer remains controversial. Chen et al. [249] showed that *TET1* and 5hmC are upregulated in epithelial ovarian cancer tissues and associated with low patient survival. They attributed this oncogenic effect of TET1 to its activation of multiple oncogenic pathways, including the immunoregulatory network centered on casein kinase II subunit α [249]. In contrast, Duan et al. [325] suggested that TET1 has a tumor suppressor effect in ovarian cancer because TET1 inhibits typical Wnt/β‐catenin signaling by activating the signaling inhibitors DKK and secreted Fzd receptor protein 2, which suppresses EMT and metastasis of the cancer cells. Furthermore, TET3 can inhibit ovarian cancer because it blocks TGFβ1 (the most classical and frequently used EMT‐inducer)‐induced EMT through upregulation of miR‐30d, thus acting as a tumor suppressor [326].

##### 5.4.1.10 Cholangiocarcinoma

The progression of cholangiocarcinoma is highly correlated with *TET1* expression. Bai et al. [251] showed that the mRNA and protein levels of TET1/3 in cholangiocarcinoma tissues were markedly higher than those in normal bile duct tissues and were positively correlated with cholangiocarcinoma malignancy. Mechanistically, TET1 enhances cell proliferation and inhibits apoptosis in cholangiocarcinoma through epigenetic regulation of the Notch and EGFR signaling pathways [251]. In contrast, *TET1* expression was decreased in intrahepatic cholangiocarcinoma [327]. This is attributed to elevated miR‐191 in intrahepatic cholangiocarcinoma, which reduces the expression of *TET1*, leading to a decrease in the anticancer activity of p53, which ultimately promotes the development of intrahepatic cholangiocarcinoma [327].

Briefly, TET proteins are extensively involved in the development of various cancers, mainly by reprogramming the DNA methylome and transcriptome landscapes. The absence of its catalytic product, 5hmC, is widely regarded as an epigenetic marker of cancer and is used as a molecular biomarker for cancer diagnosis and prognosis [247, 328]. However, the function of TET in human cancer remains unclear. In most cases, TET is defined as a cancer suppressor; however, it has also been shown that TET (especially TET1) promotes cancer formation. This switch between oncogenesis and oncosuppression may be attributed to the heterogeneity of different tumor microenvironments. Yang et al. [329] suggested that TET1 functions as a tumor suppressor under normoxia and as an oncogenic agent under hypoxia. Mechanistically, under hypoxic conditions, TET1 promotes casein kinase II subunit beta expression by acting as a coactivator of HIF‐1α to stabilize the transcriptionally active complex of HIF‐1α/p300. Consequently, casein kinase 2 activates the protein kinase B/glycogen synthase kinase 3 beta signaling pathway to promote oncogenesis [329]. This elucidation effectively addresses the heterogeneity of the roles of TET1 in different cancers. In conclusion, an in‐depth understanding of the role of TET in tumorigenesis and cancer progression could provide new potential therapeutic targets for cancer that would be of great significance to patients.

#### 5.4.2 TET and Hematopoietic Malignancies

TET proteins were initially identified in 2003 as leukemia‐associated proteins of unknown function [12, 13], but it was not until 2009 that Tahiliani et al. [6] identified TET as a hydroxylase that oxidizes 5mC substrates for demethylation. This established the first decisive evidence for the involvement of TET in disease development.

Since then, several studies have observed the disruption of the DNA landscape in almost all types of hematopoietic malignancies, which has become a hallmark of various types of hematologic cancers, including myeloid and lymphoid malignancies [16, 72, 330]. Loss‐of‐function mutations in the *TET* gene (primarily *TET2*) are thought to be major disruptors of the DNA methylation landscape in hematopoietic malignancies, leading to aberrant hematopoietic stem cell self‐renewal [331].

*TET2* is the most highly expressed *TET* gene in hematopoietic tissues and is essential for hematopoietic stem and progenitor cell (HSPC) homeostasis [332, 333]. In the hematopoietic system, clonal hematopoiesis of indeterminate potential (CHIP), also known as clonal hematopoiesis or age‐related clonal hematopoiesis, is a precancerous state. Mutations in *TET2* are important in driving CHIP [334, 335, 336]. Corresponding to this result, among the *TET* family genes, *TET2* mutations are common in hematologic cancers, which are found in myeloid and lymphoid malignancies [331, 337, 338]. *TET2* mutations and loss of function have been reported in myeloid malignancies, including myeloproliferative neoplasm (MPN), acute myeloid leukemia (AML), secondary AML, myelodysplastic syndrome (MDS), and chronic myelomonocytic leukemia (CMML) [339, 340, 341, 342, 343, 344, 345]. Among the lymphoid malignancies, *TET2* mutations are observed in approximately (2%) of B‐cell lymphomas and (12%) of T‐cell lymphomas [346]. Unlike those in *TET2*, mutations in *TET1* and *TET3* are rarely observed in hematological malignancies; however, these two genes are involved in the development of hematological cancers through expression regulation [347, 348].

**FIGURE 5:**
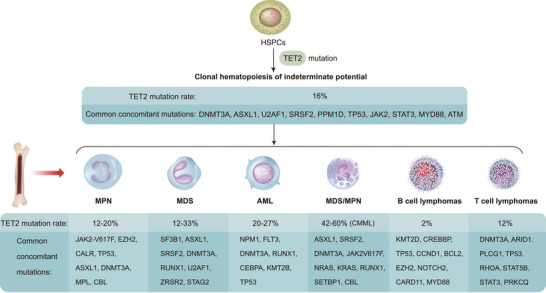
*TET2* and its concomitant mutations cause HSPCs to enter the clonal hematopoiesis of indeterminate potential (CHIP, a precancerous lesion) state. *TET2* has different mutation rates in CHIP and different hematologic cancers. However, mutations in *TET2* alone are not sufficient to cause cancer but are driven by co‐mutations in *TET2* and multiple genes, leading to the development of hematologic cancers, which include myeloid neoplasms, MPN, MDS, AML, MDS/MPN, B cell lymphomas, and T cell lymphomas. The high‐frequency and important concomitant mutations in these cancers are further discussed. Abbreviations: HSPC, hematopoietic stem and progenitor cell; TET, ten‐eleven translocation; CHIP, clonal hematopoiesis of indeterminate potential; MPN, myeloproliferative neoplasm; MDS, myelodysplastic syndrome; AML, acute myeloid leukemia.

In these hematologic malignancies, mutations in *TET2*, a tumor suppressor, drive tumorigenesis and development; however, insufficient mutations in a single gene cause the transformation of hematologic malignancies [349]. *TET2* mutations enhance hematopoietic stem cell (HSC) self‐renewal, which subsequently drives the development of various hematologic malignancies together with mutations in DNMT3A, JAK2, additional sex combs‐like 1, serine and arginine rich splicing factor 2, splicing factor 3b subunit 1, nucleophosmin 1, fms‐related receptor tyrosine kinase 3, B cell lymphoma 6 (Bcl6), tumor protein p53, and KRAS [331, 333, 350‐352]. *TET2* exhibits varying mutation rates in CHIP and different hematologic cancers [76, 342, 343, 345, 346, 353, 354]. The presence of *TET* mutations, in conjunction with mutations in different genes, plays a critical role in determining the type of malignant tumor (Figure 5) [342, 345, 346, 351, 354‐360]. Mice with a deletion of *TET2* combined with Ras homolog gene family member A (RHOA) G17V or DNMT3A R882H mutations develop mature T‐cell lymphoma [361, 362]. In contrast, germinal center (GC)‐specific overexpression of the transcription factor Bcl6 cooperates with *TET2* deletion to generate mature B‐cell lymphomas [351]. Interestingly, the coexistence of epigenetic mutations (DNMT3A and *TET2*) has been detected in patients with almost all types of hematopoietic malignancies, suggesting the potential for targeted epigenetic therapies [331]. The possibilities of such therapies are discussed in detail later in this review. Recent studies have shown that hematological disorders with *TET2* mutations can exacerbate other diseases. In patients with clonal hematopoiesis, *TET2* mutations exacerbate the progression of chronic liver disease, heart failure, atherosclerosis, chronic kidney disease, and other diseases [353, 363‐367]. These studies advance our understanding of the role of TET2 in hematologic cancers and provide fresh insights into the subtle crosstalk of disease across different organs.

The mechanisms by which *TET* mutations mediate the development of hematological malignancies have only been partially elucidated. This is largely attributed to the absence of TET2 catalytic function and the interaction between different mutated genes. High‐frequency mutations in *TET* were found in lymphoid and myeloid malignancies, with approximately 67% of these mutations located in the catalytic structural domain [350]. Consistent with this result, deletion of *TET2*, both *TET2^(±)^* and *TET2^(−/−)^*, resulted in a marked reduction in the level of 5hmC in their genomic DNA in vivo [76]. In myeloid malignancies, TET2 acts as a key tumor suppressor. The absence of *TET2* not only increases the proliferative capacity of HSC but also causes cell differentiation toward monocyte/granulocyte lineages [76]. TET2 is also a downstream target of other mutated genes that mediate the development of myeloid malignancies. Mutations in the NADP‐dependent IDH genes IDH1/2 convert isocitrate to 2‐HG instead of α‐KG [331, 368]. Although 2‐HG is a competitive inhibitor of α‐KG‐dependent enzymes, including TET family proteins, it also inhibits TET2 activity [331, 368]. Therefore, IDH1/2 mutations may have effects similar to those of *TET2* mutations. In addition, there is a physical interaction between WT1 and TET2, and WT1 loss of function has a similar effect to TET2 deficiency [331, 369, 370]. The IDH1/2‐TET2‐WT1 pathway in myeloid malignancies explains the mutual exclusivity of IDH1/2, WT1, and *TET2* mutations. Mutations in *TET2* in HSPCs have been proposed to cause DNA damage and activation of the cyclic GMP–AMP synthase‐stimulator of IFN genes pathway, which induces the production of inflammatory factors that promote AML development and human age‐related clonal hematopoiesis [333]. This is consistent with previous findings that inflammatory signals (e.g., IL‐6, TNF‐α, and IFN‐γ) accelerate *TET2* deletion‐driven leukemogenesis [371, 372].

TET2 loss of function driving lymphoid tumorigenesis is closely associated with GC [373]. GC are areas of lymphoid organs where activated B cells undergo somatic mutations and proliferate rapidly, eventually generating memory B cells or plasma cells, and where malignant tumors originate [374, 375]. TET2 plays a central role in the exit of B cells from the GC reaction, and its loss‐of‐function disrupts B cell transit through the GC, leading to GC hyperplasia, impaired class‐switch recombination, and impaired plasma cell differentiation [373]. Molecularly, *TET2* deletion leads to a reduction in enhancer 5hmC and histone acetylation, which suppresses the expression of several genes involved in GC exit and plasma cell differentiation, such as PR/SET domain 1 [373]. Interestingly, these genes and enhancers are also repressed in CREB binding protein (CREBBP)‐mutated diffuse large B‐cell lymphomas. *TET2* and CREBBP mutations are usually mutually exclusive, suggesting a synergistic or interdependent role for TET2 and CREBBP in the GC response [373]. Furthermore, TET2 deficiency drives the development of mature B‐cell malignancies, which are also attributed to activation‐induced deaminase‐mediated accumulating mutations and BCR‐mediated signaling [376]. These studies suggest that TET2‐driven lymphoma development may be the result of synergistic multi‐gene and multi‐signaling pathways.

Peripheral T‐cell lymphoma (PTCL) includes angioimmunoblastic T‐cell lymphoma (AITL), PTCL not otherwise specified, and PTCL with a TFH phenotype [377]. Mutations in both *TET2* and RHOA have been observed in up to 70% of cases [378]. Expression of RHOA G17V encoded by the RHOA mutation and *TET2* loss induces T‐cell lymphomas with features of AITL, and the proliferation of such tumor cells is dependent on the activation of inducible co‐stimulator/PI3K/mTOR signaling [361, 362, 379]. In addition, both RHOA and *TET2* mutant cells exhibited decreased FOXO1 expression and activity, and reexpression of FOXO1 reversed these defects in TET2 and RHOA cells, suggesting that FOXO1 is also a target of TET2 and RHOA [380]. Interestingly, *TET2* deletion promotes the expansion of TFH tumor cells through the CD40–CD40LG axis, leading to the development of AITL [381]. Unlike myeloid malignancies, in which IDH1/2 and *TET2* mutations are often mutually exclusive, IDH2^R172^ and *TET2* mutations are not exclusive and frequently coexist in PTCL [382]. Lemonnier et al. [383] demonstrated that IDH2^R172^ mutation produces higher levels of 2‐HG in lymphocytes compared with other IDH mutations to inhibit a variety of α‐KG‐dependent dioxygenases, which are involved in a variety of cellular functions including histone methylation, hypoxia response, and collagen maturation and are also capable of impairing lymphocyte development. This may explain the predominance of this mutation in AITL.

Notably, *TET2* mutations not only drive the development of the hematologic malignancies described previously but also mediate cellular resistance to chemotherapeutic agents such as anthracyclines and cytarabine [384]. The resistance mechanism arises from *TET2* mutations that alter the dynamics of transitions between differentiated and stem‐like states, thereby enhancing population fitness for chemotherapy [385]. These studies provide theoretical support for the treatment of chemotherapy‐resistant diseases using hypomethylation drugs.

## 6 TET‐Targeted Diagnosis and Treatment

**FIGURE 6:**
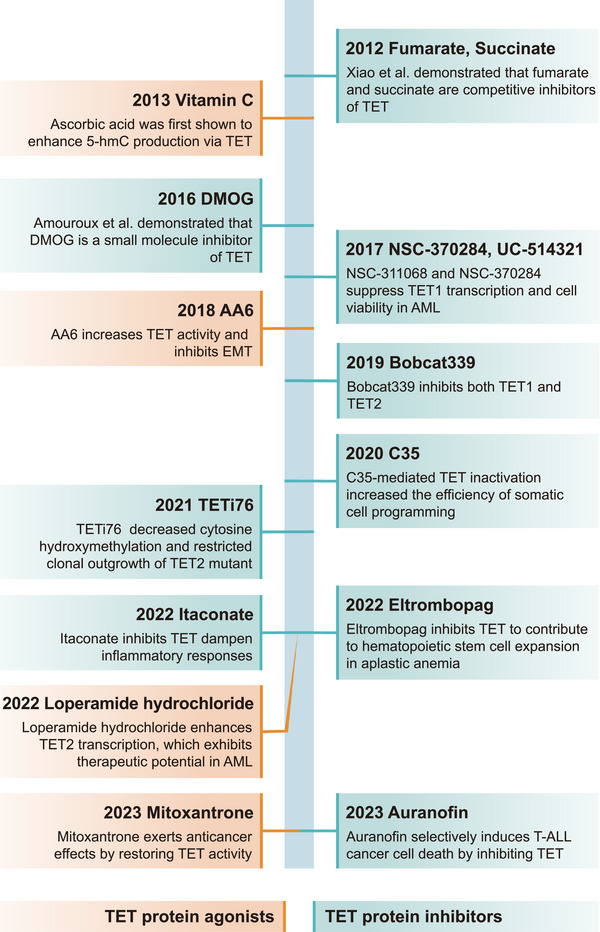
Development timeline of TET protein agonists and inhibitors. Since 2009, when TET proteins were first found to catalyze 5mC demethylation, there has been a growing recognition of the critical role of TET in physiology and pathology (especially cancer). Since then, agonists and inhibitors targeting TET have received extensive attention in clinical and basic research. Among them, vitamin C has been widely studied for its potential therapeutic role in hematologic tumors due to its safety and accessibility. In recent years, the development of agonists and inhibitors targeting TET has notably accelerated. Abbreviations: TET, ten‐eleven translocation; 5hmC, 5‐hydroxymethylcytosine; DMOG, dimethyloxallyl glycine; AA6, (S)‐2‐[(2,6‐dichlorobenzoyl) amino] succinic acid; TETi76, 2‐methelene and 4‐hydroxy; AML, acute myeloid leukemia; T‐ALL, T‐cell acute lymphoblastic leukemia.

As the molecular network of TET regulation and function unfolds, the link between 5hmC, the oxidation product of TET, and cancer is widely recognized, providing opportunities for cancer detection and management [386, 387]. Mutations and dysregulation of *TET* are closely associated with the development of a wide range of diseases, suggesting the treatment of these diseases by regulating *TET* levels. Given the unique role of TET proteins in cancer, current efforts have primarily focused on reactivating or inhibiting TET activity to treat tumors. Here, we summarize the diagnostic and prognostic values of 5hmC levels in cancer and summarize TET‐targeted therapies from the perspective of activator or inhibitor over the past decade (Figure 6), including certain of the promising therapeutic approaches currently employed in clinical and basic research.

### 6.1 Diagnostic and Prognostic Value of 5hmC Levels in Cancer

5hmC is a product of the oxidative modification of 5mC by TET [388]. Given that *TET* is frequently mutated or dysregulated in various cancers, this also suggests a close association between 5hmC and tumorigenesis. Current evidence suggests that the levels of 5hmC are generally reduced in a wide range of cancers, including glioblastomas; hematopoietic malignancies; and breast, prostate, melanoma, gastric, renal, lung, pancreatic, colon, and hepatocellular cancers, among others (Table 2) [72, 247, 389]. The reduction of 5hmC in tumors has been attributed to the following reasons: (1) mutations in *TET* resulting in a reduction in catalytic 5hmC production, which is particularly evident in various hematological cancers; (2) downregulation of *TET* expression leading to a reduction in 5hmC levels. Factors such as hypoxia, microRNA, glucose/glutamine levels, 2‐HG, and IDH mutations in tumors downregulate *TET* expression [72, 368, 390‐392]; and (3) rapid growth of tumor cells, resulting in a replication‐dependent loss of 5hmC [393]. In combination with these factors, tumor suppressor gene promoter methylation is elevated, which inhibits the binding of RNA polymerase to transcription factors, leading to transcriptional silencing and genomic instability [394]. These features suggest that 5hmC may be a valuable biomarker for cancer diagnosis and assessing prognosis [395].

The main sample sources for 5hmC testing include liquid biopsies (mainly circulating cell‐free DNA [cfDNA]) and tumor tissue samples. In clinical practice, patient‐derived cfDNA is a promising option for analyzing 5hmC levels, because cfDNA in the blood of patients with cancer can originate from tumor tissues and reflect genetic and epigenetic alterations in tumor tissues [396]. The 5hmC feature in cfDNA is a reliable and sensitive epigenetic marker, and multiple studies have extensively explored the potential applications of 5hmC levels in cfDNA and tissue samples, demonstrating its high sensitivity and specificity as a biomarker and its role in early cancer detection, cancer staging and subtype determination, and disease surveillance (Table 3) [397, 398]. Additionally, this marker is superior to conventional biomarkers in certain cancers, including colorectal and gastric cancers [399]. In addition, Kamdar et al. [400] proposed that the combined analysis of DNA methylation and hydroxymethylation in prostate cancer may enable the identification of target genes for epigenetic modifications associated with prostate cancer, which can provide new perspectives in molecular studies of cancer.

**TABLE 3:** Diagnostic and prognostic value of 5hmC in cancer.

Cancer	Sample	Number of cases	Diagnosis and forecasting	References
Esophageal cancer	cfDNA	150	Biomarkers for minimally invasive diagnosis in esophageal cancer	[401]
Gastric cancer	cfDNA	75	Biomarkers to predict cancer	[399]
Colorectal cancer	cfDNA	80	Classification of colorectal cancer; biomarkers to predict cancer	[399]
	Polyps tissue	7	Biomarkers for diagnosing colon cancer	[402]
Lung cancer	cfDNA	157	Enhanced biomarkers in lung cancer	[386]
NSCLC	cfDNA	66	Biomarkers for noninvasive diagnosis of NSCLC	[403]
Prostate cancer	cfDNA, tissue samples	165	Biomarkers of prostate cancer progression	[404]
Multiple myeloma	cfDNA	19	Noninvasive marker for studying the progression and prognosis of multiple myeloma	[405]
Hepatocellular carcinoma	cfDNA	10	Detect and monitor treatment outcomes and disease recurrence, and identify cancer types	[397]
	cfDNA	1204	Distinguish patients with early HCC from those with a history of CHB or liver cirrhosis and show superior performance over AFP	[406]
	Tissue samples	646	Prognostic marker in patients with surgically resected HCCs	[407]
Pancreatic cancer	cfDNA	7	Early detection of pancreatic cancer	[397]
PDAC	cfDNA	64	Classification of PDAC even during early‐stage disease	[408]
AML	cfDNA	115	Exhibits a higher sensitivity than MFC and molecular methods; strongly associated with clinical outcomes	[409]
AML (treatment with azacitidine combined with chemotherapy)	Bone marrow, peripheral blood	40	Changes in 5hmC after treatment are positively correlated with patient survival, with a prognostic predictive value	[410]
Pituitary neuroendocrine tumors	Plasma samples	57	The demethylation process negatively correlates with proliferation rate; potential biomarkers for tumors	[411]
Kidney cancer	Tissue samples	238	Independent prognostic marker for kidney cancer	[395]

Abbreviations: 5hmC, 5‐hydroxymethylcytosine; AFP, α‐fetoprotein; AML, acute myeloid leukemia; cfDNA, circulating cell‐free DNA; CHB, chronic hepatitis B virus infection; HCC, hepatocellular carcinoma; MFC, multiparameter flow cytometry; NSCLC, non‐small‐cell lung cancer; PDAC, pancreatic ductal adenocarcinoma.

These studies demonstrated the diagnostic and prognostic value of 5hmC assays in cancer diagnosis and prognosis. However, some challenges limit the use of 5hmC as a biomarker for clinical applications. The abundance of 5hmC in tumor tissues is influenced by various factors within the body's internal environment (e.g., vitamin C levels) [398]. Additionally, different subtypes and stages of different cancers can lead to dynamic changes in 5hmC levels. Second, blood‐derived cfDNA can have multiple origins beyond tumor tissues; thus, comprehensive cross‐tissue comparisons are required to establish highly tissue‐specific 5hmC profiles in cfDNA or to identify sources from a variety of cfDNAs using emerging technologies, such as single‐cell epigenetic assays [396]. In conclusion, with the advancements of quantitative techniques for hydroxymethylation analysis, such as hMeDIP‐seq and TAB‐Seq, that provide a favorable approach for the study of DNA hydroxymethylation modifications in tumors [93], it is reasonable to believe that exploring DNA hydroxymethylation modifications in solid and hematological tumors will facilitate the search for new tumor molecular markers and therapeutic targets that can be of immense value in the diagnosis, treatment, and prognosis of cancer.

### 6.2 TET‐Targeted Therapy

Aberrant DNA methylation is a hallmark of certain cancers [72], which also occurs in some noncancerous diseases. In recent years, researchers have designed a series of small‐molecule drugs for epigenetic modulation based on the reversibility of epigenetic marks. DNMT inhibitors have been tested in clinical trials against various cancers, and drugs, such as 5‐AZA and decitabine, have been approved by the United States Food and Drug Administration (US FDA) for clinical use [412]. This has galvanized enthusiasm for developing epigenetic regulatory drugs targeting TET in the hope of creating clinical applications by modulating TET activity and reshaping the epigenetic landscape. These include TET agonists and inhibitors (Tables 4 and 5). TET agonists are used to activate genes by enhancing 5mC oxidative activity mainly by mimicking cofactors or stabilizing protein conformation, while inhibitors act mainly by competitively blocking catalytic sites or disrupting epitope complex interactions.

**TABLE 4:** Clinical and animal studies of vitamin C as a TET cofactor in disease treatment.

Dose and treatment	Test subjects	Disease type	Epigenetic measure	Therapeutic effects	References
Oral dose of 500 mg/day ascorbate + DNMTi	Human	MDS, AML, CMML	5hmC/5mC↑	Upregulation of some viral defense genes in DNMTi naïve patients; enhancement of the biological effects of DNMTis	[413]
i.v. dose of 50–80 mg/kg/day ascorbate + DCAG (DNMTi + cytarabine + aclarubicin + G‐CSF)	Human (older adults)	AML	5hmC and TET2↑	Higher complete remission rate and better median overall survival in clinical trials	[414]
i.p. dose of 2 or 4 g/kg ascorbate + PARPis	Mice	AML	5fC and 5hmC↑	A simple injection of ascorbate (4 g/kg) or combination (2 g/kg) with PARPis promoted differentiation, slowed disease progression, and increased survival	[415]
i.p. dose of 0.5 g/kg ascorbate + anti‐PD‐L1	Mice	Renal cell carcinoma	5hmC and TET2↑	Sensitization of the tumor to anti‐PD‐L1 treatment	[416]
Providing 330 mg/L or 16.5 mg/L ascorbate in drinking water	Mice	Nerve crush	5hmC↑	Promotion of Schwann cell remyelination	[417]
i.p. dose of 500 mg/kg ascorbate	Mice	Stroke	5hmC and TET↑	Exerting neuroprotective effects after stroke (reducing infarct volume and improving motor and cognitive function); preventing post‐stroke pathophysiologic events; improving recovery	[418]
Oral dose of 0.36 g/kg/day ascorbate	Mice (*TET1^+/+^* and TET1^+/−^)	HFD‐induced obesity	5hmC↑	Improving the metabolic state; reversing adipocyte hypertrophy; reducing hepatic steatosis	[230]
i.p. dose of 4 g/kg/day ascorbate + anti‐PD‐L1	Mice	Melanoma	5hmC and TET↑	Increased tumor‐infiltrating CD8+ and CD3 cells; enhanced the efficacy of anti‐PD‐L1 immunotherapy; extended mean lifespan	[254]
i.v. dose of 1 g/kg/5 days/week ascorbate	Mice	Clear cell renal cell carcinoma	5hmC↑	Reducing the growth rate and grade of the tumor	[419]
i.p. dose of 2 g/kg/day ascorbate	Mice	Bladder cancer	5hmC↑	Slower growth and a smaller tumor burden	[420]
Providing 0.033 g/L, 0.33 g/L, or 3.3 g/L ascorbate in drinking water	Mice	Myeloid leukemia	5hmC/5mC↑	Preventing the evolution of myeloid neoplasia; increasing survival in a dose‐dependent way	[421]
High‐dose ascorbate + anti‐PD1	Mice	Lymphoma	5hmC↑	Inhibition of tumor growth and potentiation of anti‐PD1 checkpoint inhibition	[422]
i.p. dose of 0.5 g/kg ascorbate	Mice	Clear cell renal cell carcinoma	5hmC↑	Reduction of xenograft tumor weights	[423]
i.p. single dose of 4 g/kg ascorbate	Mice	AML	Increase TET2 and TET3 activity and level of 5hmC	Preventing myeloid disease progression	[424]
1% ascorbate diet; 100 mg/L of ascorbate in drinking water	Gulo^−/−^ mice; wild‐type mice	Leukemia	5hmC↑	Extended survival and prevented leukemia progression	[425]

Abbreviations: 5hmC, 5‐hydroxymethylcytosine; AML, acute myeloid leukemia; CMML, chronic myelomonocytic leukemia; DNMTis, DNA methyltransferase inhibitors; US FDA, US Food and Drug Administration; HFD, high fat diet; i.p., intraperitoneally; i.v., intravenous; MDS, myelodysplastic syndrome; PARPis, poly‐ADP‐ribosyl polymerase inhibitors; TET, ten‐eleven translocation.

**TABLE 5:** Research on TET agonists and inhibitors in disease treatment.

Type	Drug	Test subjects	Disease	Target	Therapeutic effects	References
TET protein agonists	Mitoxantrone	Leukemia cell	Leukemia	TET	Induced cell death in leukemia cells	[426]
	Loperamide hydrochloride	AML cells and mice	AML	TET	Increased 5hmC level, suppressed AML cell growth, and delayed leukemia progression	[427]
	AA6	Mice	Breast cancer‐associated lung metastasis	Increase α‐KG	Inhibited lung metastasis of breast cancer	[428]
	AA6	CMSCs, mice with type 2 diabetes	−	Increase α‐KG	Increased DNA demethylation, glucose uptake, and insulin response	[198]
	Ivosidenib	Patients with IDH1‐mutant glioma	Glioma	Increase α‐KG	Increased 5hmC and decreased tumor size	[429]
	Enasidenib	AML cells and mice	AML	Increase α‐KG	Induced cellular differentiation; survival benefit observed in mice	[430]
TET protein inhibitors	Auranofin	T‐ALL cells and mice	T‐ALL	TET1	Induced T‐ALL cell death; prolonged mice survival	[431]
	Bobcat339	Mice	Anorexia nervosa	TET3	Mitigated anorexia and elicited anxiolytic effects	[432]
	Itaconate	Mice	Inflammation	TET2	Decreased 5hmC level, reduced LPS‐induced acute pulmonary edema and lung and liver injury, and reduced LPS‐induced mouse mortality	[433]
	NSC‐370284 and UC‐514321	Mice	AML	TET1	Repressed *TET1*‐high AML progression and prolonged the median survival of TET1‐high AML mice	[434]
	UC‐514321	Medulloblastomacells	Medulloblastoma	TET1	Induced tumor cell death	[435]
	Fumarate	Sheep	−	TET1	Inhibited pregnancy and steroid hormone‐induced increases in BKCa channel current density and BKCa channel‐mediated relaxations	[436]
	Eltrombopag	Bone marrow cells from patients with aplastic anemia, leukemia cells, and mice	Aplastic anemia, leukemia	TET2	Decreased 5hmC; prevented neoplastic clonal evolution in vitro and in vivo; expanded HSPC	[437]
	DMOG	Cholangiocarcinoma cells	Cholangiocarcinoma	TET1	Decreased 5hmC level; diminished cholangiocarcinoma growth	[251]
	C35	Leukemia cells	Myeloid neoplasms	TET1/2/3	Increased the efficiency of somatic cell reprogramming	[438]
	TETi76	293T cells and mice	Leukemia	TET1/2/3	Selectively restricted the growth of *TET2*‐mutant cells; restricted the growth of *TET*‐deficient leukemia in vivo	[439]

Abbreviations: 5hmC, 5‐hydroxymethylcytosine; AA6, (S)‐2‐[(2,6‐dichlorobenzoyl) amino] succinic acid; AML, acute myeloid leukemia; AML, acute myeloid leukemia; CMSCs, cardiac mesenchymal cells; DMOG, dimethyloxallyl glycine; HSPC, hematopoietic progenitor and stem cell; LPS, lipopolysaccharides; T‐ALL, T‐cell acute lymphoblastic leukemia; TET, ten‐eleven translocation; TETi76, 2‐methelene and 4‐hydroxy; α‐KG, α‐ketoglutarate.

#### 6.2.1 TET Protein Agonists

##### 6.2.1.1 Vitamin C

Vitamin C was originally identified as an antioxidant [440]. However, in recent years, its identity as a cofactor of the TET enzyme has received increasing attention [440, 441]. Mechanistically, TET is a class of dioxygenases dependent on Fe(II) and α‐KG, with vitamin C serving as an electron donor to maintain the divalent state of Fe ions, thus regulating TET activity [441, 442, 443]. Vitamin C deficiency is common in patients with cancer [444, 445, 446, 447]. Naturally, the treatment of cancer using vitamin C supplementation has been extensively studied (Table 4).

Cellular experiments have shown that vitamin C is capable of enhancing the catalytic activity of TET enzymes in different cancer cells, including leukemia; lymphoma; breast, pancreatic, liver, colon, and thyroid cancers; and melanoma, among others, thereby promoting the oxidation of 5mC to 5hmC [448]. In vivo experiments, have primarily focused on the potential anticancer role of vitamin C in hematological tumors. This is partly attributed to the frequent occurrence of *TET2* loss‐of‐function mutations in hematological tumors [380, 449], and the reduction in TET2 activity resulting from these mutations may be possibly rescued by vitamin C supplementation. Consistent with this, several studies have proposed that vitamin C treatment mimics the effects of TET2 repair in leukemic stem cells and slows leukemia progression by enhancing TET activity and killing PARP inhibitors, whereas ascorbate (fully reduced vitamin C) depletion accelerates leukemia by reducing TET2 function [415, 424, 425]. Using TET2‐ and ascorbic acid‐deficient model systems, Guan et al. [421] showed that ascorbate directly binds to the TET2 catalytic structural domain and enhances its activity, thereby delaying myeloid tumorigenesis in mice. In a mouse model of lymphoma, high‐dose ascorbate synergistically treated with anti‐PD1 increased tumor immune recognition and potentiated the anticancer effects of anti‐PD1 immunotherapy to inhibit tumor growth [422]. In clinical trials, vitamin C has been frequently used as an adjuvant in standard chemotherapy regimens. Synergistic treatment with vitamin C and azacitidine increased the 5hmC/5mC ratio and upregulated viral defense genes in patients with myeloid tumors [413]. Vitamin C synergized with decitabine‐activated TET2 in leukemia cells, which significantly improved the overall survival of older patients with AML [414].

In addition to hematological tumors, ascorbate therapy is effective for solid cancers with low 5hmC levels. In vivo experiments have shown that ascorbic acid treatment increases TET activity, enhances lymphocyte tumor infiltration and antitumor immunity, and prolongs the lifespan of tumor‐bearing mice [254]. In addition, vitamin C increased 5hmC levels and prevented the growth of bladder cancer and clear cell renal cell carcinoma, suggesting that vitamin C acts therapeutically by increasing TET activity [419, 420, 423]. Similar to hematological cancers, vitamin C synergizes with other chemotherapeutic agents in solid tumors. Peng et al. [416] showed that vitamin C induces PD‐L1 expression and enhances the sensitivity of renal cell carcinoma cells to anti‐PD‐L1 immunotherapy by stimulating TET2 enzyme activity. In conclusion, these studies suggest that vitamin C has great potential for the treatment of solid tumors. In nontumor diseases, the application of vitamin C can play a therapeutic role in the repair of nerve damage and the alleviation of obesity and metabolic disorders by activating TET to promote an increase in 5hmC levels [230, 417, 418].

These studies highlight the therapeutic potential of vitamin C as a cofactor for TET. However, challenges remain regarding the clinical application of vitamin C. In addition to tumor suppression through the activation of TET, vitamin C can also combat cancer by exploiting redox imbalances and targeting HIF‐1 signaling in cancer cells [441]. It is unclear whether vitamin C‐activated TET plays a critical role in anticancer processes. Furhtermore, there is heterogeneity in the therapeutic concentration of vitamin C in different types of cancer cells [420], and the specific therapeutic concentration should be considered in the context of the tumor type, mode of administration, and synergistic treatment with different chemotherapeutic agents. Additionally, the antioxidant activity of vitamin C may reduce the therapeutic efficacy of killing cancer cells through oxidative mechanisms (radiation therapy with ionizing radiation and chemotherapeutic agents) [450]. In conclusion, vitamin C is a promising and inexpensive anticancer therapeutic agent that should be further explored in clinical trials.

##### 6.2.1.2 Mitoxantrone

Mitoxantrone is a synthetic anthracenedione that is primarily used for the treatment of breast cancer, prostate cancer, lymphoma, leukemia, and multiple sclerosis [451, 452]. The antitumor activity of mitoxantrone is associated with its role as an inhibitor of topoisomerase II and DNA replication and transcription [453]. Recently, Kim et al. [426] determined that mitoxantrone acts as a TET agonist and antagonizes cancer cell viability by increasing 5hmC levels in a cell line expressing low levels of the TET1 catalytic domain. This study also showed that toluidine blue O exhibits a similar effect [426]. Thus, these novel TET agonists may be useful in cancer therapy.

##### 6.2.1.3 Loperamide Hydrochloride

Opioid signaling activators also exhibit TET agonism [427]. Zhao et al. [427] showed that the opioid receptor agonist, loperamide hydrochloride, upregulated *TET2* expression and increased 5hmC levels through the activation of opioid signaling, thereby inhibiting AML cell growth.

##### 6.2.1.4 AA6, Ivosidenib, and Enasidenib

TET proteins are α‐KG‐dependent dioxygenases, and drugs may act indirectly as TET agonists by affecting α‐KG levels. The α‐KG dehydrogenase inhibitor, (S)‐2‐[(2,6‐dichlorobenzoyl) amino] succinic acid (AA6), is capable of altering the TCA cycle, leading to α‐KG accumulation and thus increasing TET activity [428]. Atlante et al. [428] showed that AA6 can play a role in the treatment of metastatic breast cancer by increasing α‐KG levels, TET activity, and 5hmC levels. Additionally, AA6 increases α‐KG and DNA demethylation levels in CMSCs in patients with type 2 diabetes, suggesting a potential benefit of AA6 for repairing damaged CMSCs in a diabetic setting [198]. IDH inhibitors modulate TET by affecting α‐KG. Although it remains unclear whether these IDH inhibitors antagonize tumor growth by targeting TET proteins, it was previously noted that their mutation decreases α‐KG and increases 2‐HG, leading to inhibition of TET activity [331, 368]. Thus, they may function as indirect TET agonists. Small‐molecule inhibitors targeting mutant IDH1/2, ivosidenib, and enasidenib are currently US FDA‐approved and have shown clinical efficacy in diseases such as AML, glioma, and cholangiocarcinoma [429, 454‐457].

#### 6.2.2 TET Protein Inhibitors

As previously discussed, TET agonists can be used for cancer therapy. In contrast, the use of TET inhibitors to further reduce TET activity in cancer cells may hold promise for treating diseases such as cancer. Although TET inhibitors have demonstrated potential therapeutic effects in a number of diseases, there are currently no TET inhibitors used in clinical settings, and they have been used primarily as effective silencing tools in experimental studies.

##### 6.2.2.1 Auranofin

Auranofin is a gold‐containing compound that has been used in the treatment of rheumatoid joints for the last 40 years [458, 459]. Auranofin exhibits good antitumor and anti‐infection therapeutic effects [458, 459, 460]. Recently, Chen et al. [431] found that Auranofin inhibits the catalytic activity of TET1 by competing with its substrate to bind to TET1, thereby inducing T cell acute lymphoblastic leukemia (T‐ALL) cell death and inhibiting T‐ALL progression [431]. This provides new mechanistic insights into T‐ALL therapy and presents a potential small‐molecule therapeutic agent.

##### 6.2.2.2 Bobcat339

Bobcat339, a cytosine‐based lead compound discovered by Chua et al. [461] in 2019, was initially reported to have potent inhibitory functions against TET1 and TET2. However, Shi et al. [462] subsequently questioned its inhibitory effect and concluded that, in the absence of Cu(II) contamination, Bobcat339 itself has negligible inhibitory activity against TET1 and TET2. However, it is now well‐established that Bobcat339 can negatively regulate TET3 [432]. Lv et al. [432] showed that Bobcat339 destabilizes TET3 in neuronal cells to treat anorexia nervosa in a mouse model. Additionally, the advantages of Bobcat339 as a TET inhibitor have been demonstrated by the fact that it does not adversely affect cell viability and proliferation at reasonable dosages and is well tolerated in vivo [432, 463].

##### 6.2.2.3 Itaconate

Itaconate is a metabolite with anti‐inflammatory and antimicrobial properties and serves as a potential immunosuppressant and antimicrobial agent [464, 465]. Chen et al. [433] showed that itaconate inhibits TET2 catalytic activity by competitively binding to TET2 with α‐KG. As a potent inhibitor of TET proteins, itaconate reduces 5hmC levels in vivo, decreases lipopolysaccharide‐induced acute pulmonary edema and lung and liver injury, and reduces mortality in mice [433]. These studies established that itaconate might act as a TET inhibitor to suppress the inflammatory response.

##### 6.2.2.4 NSC‐370284 and UC‐514321

NSC‐370284 and UC‐514321 were identified by Jiang et al. [434] from among 20,602 chemical compounds as TET1 inhibitors. These compounds inhibit *TET1* expression by directly targeting STAT3/5, a transcriptional activator of TET1 [434]. *TET1* is highly expressed in AML, exhibiting oncogenic effects [466, 467]. Using in vivo mouse studies, Jiang et al. [434] demonstrated that NSC‐370284 and UC‐514321 considerably inhibited the progression of TET1‐hyper AML and prolonged median survival by more than three‐fold. Similarly, in medulloblastoma, *TET1* is upregulated and is considered a key enzyme that regulates tumor progression [435]. In in vitro studies, UC‐514321 induced cell death in medulloblastoma cells and showed a response only in tumor cell lines exhibiting high *TET1* expression [435]. Therefore, NSC‐370284 and UC‐514321 may function as TET1 inhibitors, offering therapeutic implications for diseases characterized by abnormal TET1 levels.

##### 6.2.2.5 Fumarate and Succinate

Fumarate and succinate are intermediates in the TCA cycle and typically [468]. However, mutations in the TCA cycle enzymes fumarate hydratase and succinate dehydrogenase result in an abnormal accumulation of fumarate and succinate [469]. Ultimately, fumarate and succinate act as competitors for α‐KG and inhibit the activity of α‐KG‐dependent dioxygenases, including TET family proteins [469, 470]. However, fumarate and succinate are predominantly present as procancer factors [470, 471]. Currently, fumarate and succinate are widely used as pharmacological tools to explore TET‐mediated demethylation [436, 470].

##### 6.2.2.6 Eltrombopag

Eltrombopag is a nonpeptidyl thrombopoietin receptor agonist approved by the US FDA for the treatment of aplastic anemia [437]. In the presence of divalent iron, eltrombopag diffuses into the nucleus and binds specifically to the TET2 catalytic structural domain, thereby inhibiting TET2 protein function [437]. Guan et al. [437] showed that eltrombopag treatment mimicked the loss of TET2, allowing the expansion of HSPC, which partially resolved its efficacy in aplastic anemia. This ideal regulatory mechanism has a remarkable therapeutic scope.

##### 6.2.2.7 Dimethyloxallyl Glycine

Dimethyloxallyl glycine (DMOG) is a small molecule inhibitor of dioxygenase [54, 472]. DMOG treatment of breast cancer cells markedly reduced the expression of *TET1/2/3* and human leukocyte antigen‐G; high expression of the latter is essential for tumor cells to avoid immune recognition and destruction [473]. The application of DMOG to cholangiocarcinoma cells also notably reduced *TET1* expression and tumor cell growth [251]. Inhibition of TET by DMOG during embryonic development has been used to explore the mechanism of genome‐wide demethylation that zygotes undergo after fertilization [54]. Therefore, DMOG, a TET inhibitor, can play a therapeutic role in several diseases and serve as a good tool for future studies.

##### 6.2.2.8 C35

C35 is a TET inhibitor screened by Singh et al. [438] from a pool of 265,000 compounds that target all members of the TET family. This TET inhibitor is characterized by its ability to selectively target TET catalytic activity without eliminating the TET complex and can, thus, be used as an inhibitor for studying the nonenzymatic activity of TET [438]. Singh et al. [438] found that this inhibitor reduced genome‐wide 5hmC labeling and promoted somatic cell reprogramming by activating the bone morphogenetic protein/drosophila mothers against decapentaplegic protein/inhibitors of differentiation signaling pathway. This study suggests that C35 can be used as a tool to improve our understanding of the different roles of catalytic and noncatalytic TET structural domains in somatic cell reprogramming.

##### 6.2.2.9 TETi76

2‐Methelene and 4‐hydroxy (TETi76) is a competitive TET inhibitor designed and synthesized by Guan et al. [439] This TET inhibitor is designed to mimic 2‐HG and is more suitable as a TET2 inhibitor than natural 2‐HG [439]. TETi76 has been shown to selectively restrict the growth of *TET2* mutant cells in vitro and limit the development of TET2‐deficient leukemia in vivo, extensively decreasing the tumor load [439]. Thus, it has the potential to be used as a drug for targeting *TET2* mutant tumors.

Although epigenetic regulatory drugs targeting TET have shown great potential and a variety of drugs have been discovered that can affect TET activity, the widespread action of TET on cells throughout the body and the fact that most of them are not TET‐targeting drugs have limited the clinical application of the drugs. Currently, it is mainly vitamin C in combination with other chemotherapeutic drugs that are carried out in animals and clinics. In the future, the combination of TET‐targeted drugs with other chemotherapeutic agents is a promising direction, and we need to promote precise TET‐targeting strategies to improve delivery efficiency and reduce off‐target risk.

## 7 Practical Challenges in the Clinical Application of TET‐Targeted Therapy

TET proteins play key roles in mammalian development and the development of several diseases. Drugs that modulate TET, whether agonists or inhibitors, have great potential and are therefore intriguing. However, translating TET‐targeted therapies into clinical applications remains challenging.

First, although *TET2* mutations are common across all types of hematopoietic malignancies, *TET2* mutations alone are not sufficient to develop hematopoietic malignancies. These malignancies result from co‐mutations in multiple cancer genes [331]. Understanding the synergistic effect of these concurrent mutations with *TET2* mutations to induce various types of hematopoietic malignancies requires further studies before drugs targeting TET2 can be applied. Second, there is heterogeneity in the roles of TET proteins in the development of different cancer types. For example, TET1 acts as a tumor suppressor in colon and pancreatic cancers [244, 296], but as an oncogenic factor in ovarian cancer, AML, and medulloblastoma [249, 435, 466]. In the case of the former type of cancers, restoration of the enzymatic activity of TET is an effective therapeutic strategy, whereas inhibition of the enzymatic activity of TET is required in the latter. In addition, different cancer subtypes as well as different stages of cancer progression may influence the role of TET. Therefore, cancer treatment strategies for TET rely on a combination of factors. Notably, the therapeutic effects of drugs based on TET therapy, except for vitamin C, are currently being explored at the animal and cellular levels but have not been adequately investigated in clinical studies. This limitation may be partly attributed to the fact that these epigenetic modulatory drugs exert extensive side effects throughout the body, rather than exclusively acting on the target organ when they enter the body. Global epigenetic perturbations may disrupt imprinted gene regulation, causing tissue‐specific toxicity and potentially exacerbating complications, which poses a significant challenge for their clinical application.

In conclusion, extensive basic research targeting TET should be conducted before the clinical application of TET‐targeted therapy. Considering that TET is a key element of the extensive and complex regulatory network in the human body, Pandora's box surrounding the reconstruction of the DNA methylation landscape remains largely unknown.

## 8 Conclusions and Perspectives

Since 2009, when TET proteins first gained prominence in the field of epigenetics, researchers have considerably advanced our understanding of TET in pathology and pharmacology through a multitude of fascinating experimental approaches. Disturbances in DNA methylation dynamics are closely associated with the development of various diseases, which is particularly evident in hematopoietic malignancies. Notably, although mutations in the *TETs* are less common in solid malignancies, in most solid cancers, the TET enzyme is recurrently regulated and 5hmc levels are disrupted; thus, DNA methylation and demethylation mechanisms are key regulators of cancer development. As sorted out earlier, there are conflicting reports on *TET1/2/3* expression and activity in solid tumor tissues. This discrepancy may stem from many confounding factors such as cancer tissue type, tumor stage, differentiation status, the microenvironment, and so on. The unique role of TET proteins in different tissues and cell types remains poorly understood. Future studies could utilize techniques such as single‐cell RNA sequencing and single‐cell transposase‐detectable chromatin sequencing methods to map the tissue‐specific expression and dynamic regulatory networks of TET proteins.

In addition, several interesting questions in this area need to be further investigated. Why are mutations in *TETs* more prevalent in hematologic cancers, whereas relatively few *TET* mutations are detected in solid malignancies? What are the potential reasons for this discrepancy? Another question worth exploring is why *TET2* mutations are so prominent in human hematopoietic malignancies, whereas mutations in *TET1* or *TET3* are uncommon. Could this be related to the high expression of *TET2* in HSPCs or its more important role in 5mC oxidation? In addition, the interactions between TET proteins and transcription factors still need to be further investigated, especially the mechanism of interaction between their catalytic structural domains and unstructured N‐terminal regions. A deeper understanding of these interactions will help reveal the mechanism of action of TET enzymes in cancer and how they regulate gene expression. Future studies should be conducted on the multimodal and multilevel effects resulting from the dysregulation of the catalytic and noncatalytic functions of TET1/2/3. As we gain a better understanding of the function and mechanism of action of TET proteins, especially in the context of diseases such as cancer, these studies will provide new opportunities and challenges for developing TET‐targeted therapies.

In this review, we examine the structure and biological functions of TET proteins, emphasizing their role in disease pathogenesis, as well as the cutting‐edge research on TET‐targeted drug therapy. Epigenetic drugs that modulate TET have demonstrated exciting potential for the treatment of diseases, especially cancer. The combination of these drugs with other chemotherapeutic agents for cancer treatment has the potential to enhance their antitumor effect, which represents one of the most promising approaches in cancer treatment. The application of these unique epigenetic regulatory drugs requires a deeper understanding of the role of TET in disease pathogenesis. In conclusion, we anticipate that these breakthroughs in the field will help improve the treatment of most diseases and provide hope to patients.

## Author Contributions

T. L., Z. N., D. L., and X. X. conceived the review. J. L., X. N., L. M., L. H., and T. S. drafted and wrote the manuscript. Y. F., B. Z., Z. Z., and C. G. participated in part of the writing. T. L., Z. N., D. L., and X. X. reviewed and edited the manuscript. All authors approved the final version of the manuscript.

## Ethics Statement

The authors have nothing to report.

## Conflicts of Interest

The authors declare no competing interests.

## Data Availability

The authors have nothing to report.
